# Neuromedin B identified as a therapeutic target for atopic dermatitis: evidence from Mendelian randomization and PCR validation

**DOI:** 10.3389/fmed.2025.1660249

**Published:** 2025-11-19

**Authors:** Lingling Zhang, Yuxu Yao, Chunxi Ke, Xiaolin Bu, Qingqing Jiao, Jiang Ji

**Affiliations:** 1 Department of Dermatology, Gongli Hospital of Shanghai Pudong New Area, Shanghai, China; 2 Department of Dermatology, The Second Affiliated Hospital of Soochow University, Suzhou, China; 3 Central Research Laboratory, The First Affiliated Hospital of Soochow University, Suzhou, China; 4 Department of Dermatology, The First Affiliated Hospital of Soochow University, Suzhou, China

**Keywords:** atopic dermatitis, Mendelian randomization, Neuromedin B, pruritus, RT-qPCR, druggable genes

## Abstract

**Introduction:**

Atopic dermatitis (AD) is a long-standing inflammatory dermatosis marked by intense itching and immune imbalance. Despite recent advances in targeted biologic therapies, limitations in efficacy and cost highlight the urgent need for novel therapeutic targets.

**Methods:**

We employed Mendelian randomization (MR) by combining genome-wide association studies (GWAS), expression quantitative trait loci (eQTL), and protein QTL (pQTL) datasets to identify causal druggable genes associated with AD. To enhance the validity of causal inference, we further utilized colocalization and summary-data-based MR (SMR) techniques. We validated the expression of five prioritized genes using reverse transcription quantitative PCR (RT-qPCR), performed on RNA extracted from the peripheral blood of AD patients and healthy controls.

**Results:**

The MR approach revealed 32 candidate genes with potential druggable properties linked to AD, with 12 showing strong colocalization signals (posterior probability of hypothesis 4 (PP.H4) > 0.8). The pQTL analysis indicated that increased plasma NMB levels were associated with a heightened risk of AD (OR = 1.18, *p* = 3.29 × 10^-8), a conclusion further corroborated by SMR analysis. RT-qPCR confirmed significantly elevated expression of *NMB, IL2RA, IL1RL1, and PRKCQ* in the peripheral blood samples of AD patients. Additionally, MR studies demonstrated that NMB was associated with bullous pemphigoid and urticaria.

**Conclusion:**

Integrative MR and PCR validation across Icelandic, Finnish and Chinese samples nominates NMB as a candidate AD target. These preliminary, multi-ancestry signals now require replication in large, population-matched cohorts before any therapeutic translation.

## 1 Introduction

Atopic dermatitis (AD) is a chronic and recurrent dermatological disorder characterized by pruritic erythema, significantly impairing patients’ quality of life (1). Although the precise etiology and pathogenesis of AD remain elusive, current evidence implicates the activation of interleukin (IL)-13 and IL-4 inflammatory pathways, as well as epidermal barrier dysfunction, in disease development (2, 3). Clinical guidelines recommend oral or topical glucocorticoids, phototherapy, and systemic immunomodulatory agents as effective treatments for AD (4). Recently developed biologics have shown therapeutic promise; however, their high costs pose a considerable economic burden on patients (5). Despite advancements, controlling pruritus and preventing recurrence in AD remain formidable challenges, underscoring the need for novel therapeutic targets to enhance disease management.

Mendelian randomization (MR) analysis leverages genetic polymorphisms to assign individuals to control or experimental groups in a manner analogous to randomized controlled trials, enabling the investigation of causal relationship between exposure and outcome. In Mendelian randomization analyses, genetic variants such as single nucleotide polymorphisms (SNPs) that correlate with exposure traits are employed as instrumental variables (IVs) to estimate causal effects. Compared to observational studies, MR analysis mitigates the influence of confounding factors and reverse causality, and it has been widely applied in various conditions, including cardiovascular, autoimmune, and metabolic diseases (6–8).

Druggable genes are those that encode proteins potentially amenable to pharmacological intervention or that interact with approved drugs. Importantly, a portion of druggable genes falls within well-characterized gene families that closely resemble existing pharmacological targets. Expression quantitative trait loci (eQTLs) represent genetic variants that regulate gene expression levels, thereby linking genotype to transcriptional activity. In druggable genome-wide MR analysis, cis-eQTLs located near druggable genes serve as IVs to assess the causal effects of these genes on disease risk.

Building on this framework, we performed MR, colocalization, Protein quantitative trait loci (pQTL), and Summary data-based Mendelian randomization (SMR) analyses to identify potential therapeutic targets for AD, and further validated the expression of selected genes using reverse transcription quantitative PCR (RT-qPCR) in patient blood samples to enhance biological interpretability of the findings.

## 2 Methods

### 2.1 Mendelian randomization analysis of druggable genes

#### 2.1.1 Study design

**Figure 1:**
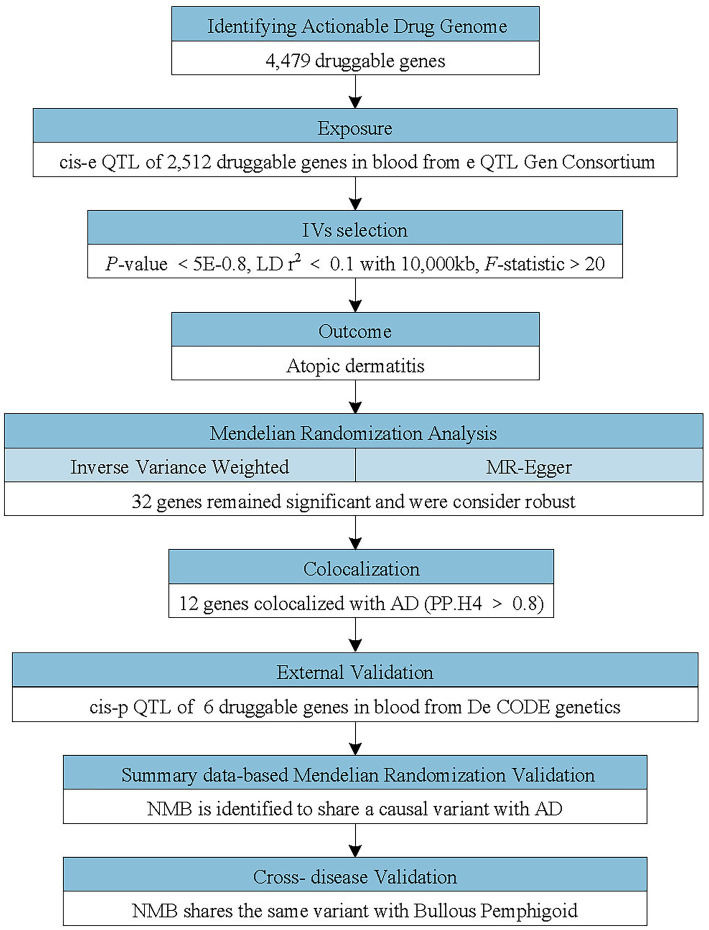
Flowchart of the study design. The study applied a multi-step Mendelian randomization (MR) strategy to identify and validate druggable genes associated with atopic dermatitis (AD). *Cis*-expression quantitative trait loci (*cis*-eQTLs; genetic variants located near the target genes affecting their expression) were used as instrumental variables (IVs) to evaluate causal effects on AD. Significant genes identified by MR underwent colocalization analyses and were further cross-validated using cis-protein quantitative trait loci (cis-pQTL) and summary data-based Mendelian randomization (SMR) analyses. Neuromedin B (NMB) was identified as a potential causal gene and additionally assessed in other pruritic conditions.

This study sought to recognize potential drug targets for AD. To explore causality between druggable genes and AD, we first applied MR analysis using cis-eQTLs in blood as instrumental variables and AD GWAS data as the outcome reference. Subsequently, we implemented sensitivity analyses to guarantee the robustness of MR analysis. Secondly, we conducted colocalization analysis for druggable genes which have notable MR results. Thirdly, pQTL analysis of druggable genes was implemented to verify the effect on AD. Lastly, SMR analysis was implemented to confirm the druggable genes that had positive results in the previous analysis. Based on the identification of Neuromedin B (NMB) as a neuropeptide associated with itch sensation, we extended the MR analysis to evaluate its potential role as a therapeutic target for additional pruritic conditions such as BP and psoriasis. Figure 1 delineates the steps of this study in detail. This research exclusively utilized publicly accessible data. To ensure methodological transparency, the study adhered to the principles of the STROBE-MR reporting standards for Mendelian randomization (9).

#### 2.1.2 Data sources

The eQTL database included genetic variants associated with gene expression levels, we obtained cis-eQTL data in the blood for druggable genes from the eQTLGen Consortium^1^, of which most individuals are European descent.

The study conducted by Ferkingstad E et al. had identified pQTL about 4,719 plasma proteins in 35,559 Icelanders (10). The data was available on the deCODE genetics^2^. We obtained the pQTL of druggable genes from this study.

The outcome variable datasets were gathered from the FinnGen consortium^3^. The continuously updated FinnGen GWAS database incorporates extensive data and provides open access. The AD dataset included 421,381 individuals (26,905 cases and 394,476 controls). The Bullous pemphigoid dataset included 452,508 individuals (609 cases and 451,899 controls). The psoriasis vulgaris dataset included 444,563 individuals (7,143 cases and 437,420 controls). Participants in these datasets were predominantly of European ancestry.

All data utilized in this study are publicly accessible and detailed in Supplementary Table S1.

Finan et al. have identified 4,479 druggable genes (11). Of these druggable genes, 1,427 encode drug targets, 682 encode proteins that react with drug molecules, and 2,370 are members of important druggable gene families (Supplementary Table S2).

#### 2.1.3 Selection of IVs

In MR analysis, choosing IVs is a crucial step, IVs must display a robust correlation with the exposure and remain independent of any confounding factors. Horizontal pleiotropy testing ensures that instrumental variables affect the outcome solely through the exposure, ruling out any alternative pathways. Several quality controls were performed in selection of SNPs from the cis- eQTL data for each druggable gene: SNPs with *p* < 5.0 × 10^−8^ were included to ensure strong association with druggable genes; Independent variants were selected by excluding SNPs in high linkage disequilibrium (LD), defined as *r*^2^ ≥ 0.1, within a ±10 Mb genomic region; incompatible SNPs between the druggable genes and AD were deleted; SNPs with an F-statistic below 10 were omitted to mitigate mild instrument bias.

#### 2.1.4 MR analysis, sensitivity analysis

The R software (version 4.2.2), R Studio software (version 8.15), R package “TwoSampleMR” (version 0.5.6) and R package “coloc” (version 5.1.0.1) were used for data analysis.

If IVs contained only one SNP, Wald ratio method was used for MR analysis. If the IVs included two or more SNPs, the inverse variance weighted (IVW) method was used as the primary estimator, supplemented by MR-Egger, weighted median, simple mode, and weighted mode approaches (Supplementary Table S4). Ignoring the possible heterogeneity, MR analysis was mainly based on IVW due to the conservative and crude characteristics, the others mode supplemented it. Ultimately, false discovery rate (FDR) corrections were employed to ascertain meaningful MR outcomes in multiple tests.

Tests for heterogeneity and tests for horizontal pleiotropy are included in sensitivity analysis. Heterogeneity is defined as the distinctions between IVs, and a *p*-value of less than 0.05 in the Cochran’s Q test suggests that heterogeneity exists. A significant MR-Egger intercept (*p* < 0.05) implied the presence of directional pleiotropy and a possible violation of MR assumptions. These assessments are now supplemented with MR-PRESSO and leave-one-out analysis for every MR estimate.

#### 2.1.5 Colonization

For the druggable genes demonstrating valuable MR outcomes, colocalization analysis was performed to analyze the shared causal variant of druggable genes and AD. The posterior probability of PP. H4 represents the possibility that cis-eQTL and outcome share a causal variant. In this study, we considered PP. H4 values above 0.8 as strong evidence supporting colocalization, and the genes colocalized with AD can be used as potential targets for additional study.

#### 2.1.6 pQTL

We conducted MR analysis using pQTL data to examine the relationship between protein levels encoded by druggable genes and AD, focusing on genes that significantly colocalize with the condition. IVs were selected based on established screening criteria, FDR corrections were utilized for detecting significant MR results, and the intercept of MR-Egger regression was utilized to evaluate potential pleiotropy.

#### 2.1.7 SMR

Summary-level statistics from GWAS and eQTL datasets were integrated in the SMR analysis to detect potential overlaps between gene expression regulation and phenotypic traits. The heterogeneity in dependent instrument (HEIDI) test was utilized to evaluate pleiotropy, with a *p-*value of less than 0.05 indicating the existence of pleiotropy. The SMR software (version 1.0.3) was utilized for the analysis of SMR. eQTL and cis-eQTL data for druggable genes in blood were sourced from the GTEx database, while GWAS data for AD was acquired from the FinnGen consortium.

### 2.2 RT-qPCR-based validation of candidate genes in AD patient blood samples

To validate the MR findings, we selected five candidate genes (*NMB, IL2RA, IL1RL1, PRKCQ, and HLA-DRB1*) for expression analysis. These genes were prioritized based on their colocalization results (PP.H4 > 0.8), FDR < 0.05, and the strength of the observed causal effect (odds ratios deviating significantly from 1). NMB was additionally supported by pQTL and SMR analyses. The combination of statistical strength and biological relevance guided our selection for experimental validation.

#### 2.2.1 Study participants and inclusion/exclusion criteria

Eight patients with atopic dermatitis and an equal number of age-comparable healthy controls were recruited for analysis. The inclusion criteria for AD patients were: age between 18 ~ 75 years, diagnosis of AD according to the 2014 American Academy of Dermatology criteria, disease duration of ≥1 year, and the ability to read and understand the informed consent and willingness to sign it. Exclusion criteria included the use of immunosuppressants or corticosteroids within the past 30 days, the presence of tumors or liver/kidney dysfunction, pregnancy or lactation, and a history of psychiatric disorders. The study received ethical clearance from the Gongli Hospital Ethics Committee (GLYY1s2023-028), and all enrolled participants provided signed informed consent.

### 2.3 RNA extraction, RT-qPCR, and statistical analysis

Blood samples from the participants were immediately stored at −80 °C after collection to preserve RNA integrity. Total RNA was extracted from whole blood using a silica membrane-based spin column method with a Tissue/Cell Total RNA Extraction Kit (Beyotime, BYT430, China). The quality of the extracted RNA was measured using a NanoDrop spectrophotometer, and RNA integrity was confirmed by agarose gel electrophoresis. To generate complementary DNA (cDNA), total RNA was reverse transcribed using the HiScript® kit (Vazyme, China) following the manufacturer’s protocol: 25 °C for 5 min, 50 °C for 15 min, and 85 °C for 5 s.

Using AceQ® SYBR Green Master Mix (Vazyme, Nanjing), qPCR amplification was performed under the following conditions: pre-denaturation at 95 °C for 5 min, then 44 cycles comprising 95 °C for 10 s and 60 °C for 30 s. Specific primers were designed for five target genes (*NMB, IL2RA, IL1RL1, PRKCQ, and HLA-DRB1*). *GAPDH* was used as the reference gene for normalization. The relative expression levels of the target genes were calculated using the 2^−ΔΔCt^ method. Primer information is provided in Supplementary Table S10.

All statistical analyses were performed in GraphPad Prism (v10.1.2). Given the limited sample size, differences between AD patients and control subjects were evaluated using the non-parametric Mann–Whitney *U* test. A two-tailed *p*-value below 0.05 was interpreted as statistically significant.

## 3 Results

### 3.1 Identification of causal genes for AD by MR

#### 3.1.1 Selection of IVs

A total of 37,672 SNPs that fulfilled the selection criteria were retained as instrumental variables for 2,007 genes with therapeutic potential (see Supplementary Table S3). All selected instruments exhibited F-statistics exceeding 10, suggesting adequate instrument strength and minimizing the risk of weak instrument bias.

#### 3.1.2 Mendelian randomization

We applied the inverse variance weighted (IVW) method as the primary estimator and identified 32 druggable genes whose genetically predicted expression was significantly associated with AD (FDR < 0.05). To guard against horizontal pleiotropy, we subsequently ran MR-Egger, weighted median, simple mode, weighted mode and MR-PRESSO together with leave-one-out diagnostics for every exposure–outcome pair (Supplementary Table S4). According to Cochran’s Q statistic, no significant heterogeneity was detected among instrumental variables used for these associations. Additionally, a notable gene exhibiting pleiotropy, as indicated by MR-Egger regression, was deleted (Supplementary Table S4). Therefore, after adjusting for pleiotropy, we confirmed the association for 32 of these genes.

#### 3.1.3 Colocalization

**Figure 2:**
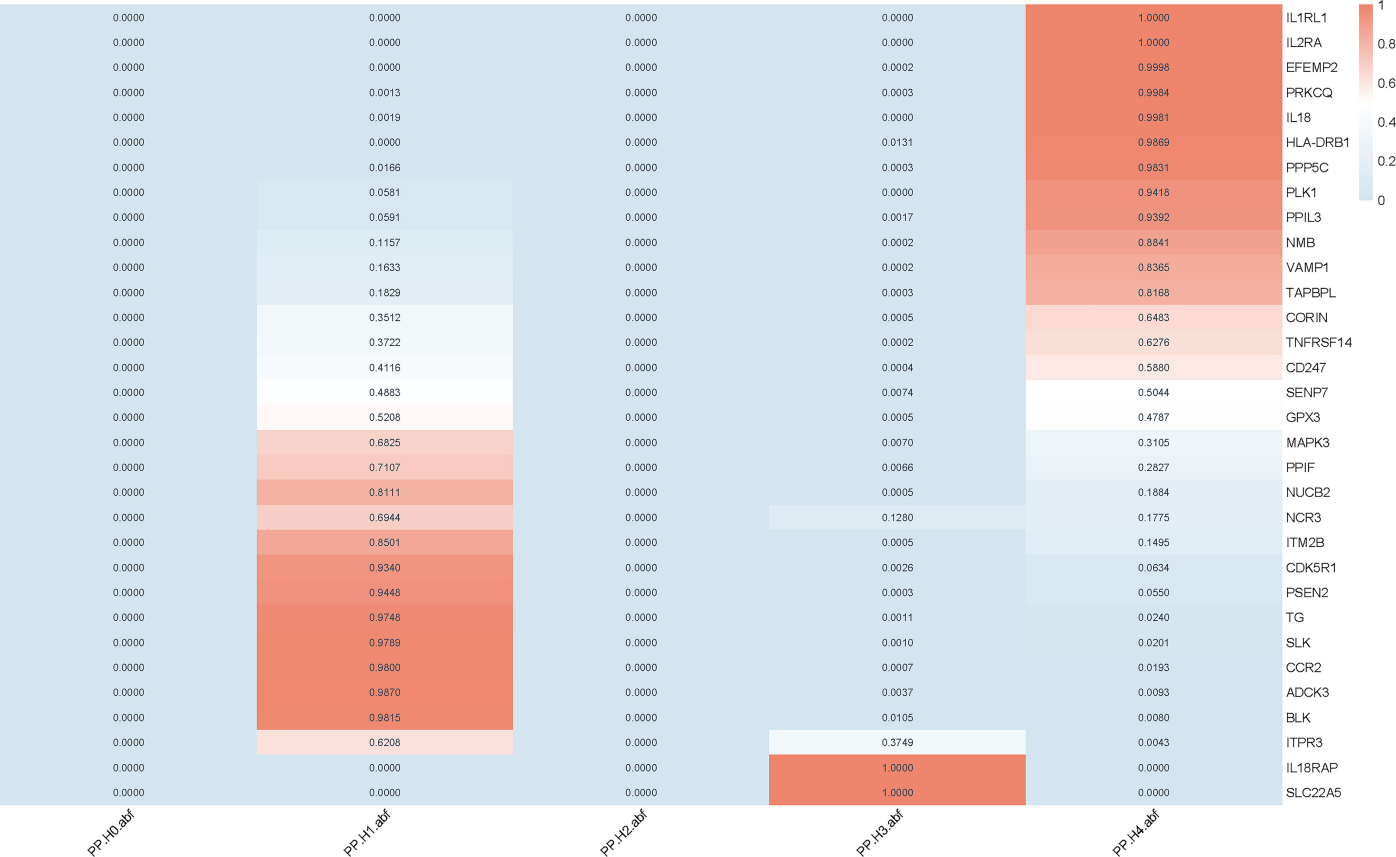
Colocalization analysis of druggable genes associated with AD. Colocalization analysis was performed for 32 druggable genes that showed significant causal associations with AD in the MR analysis. Posterior probabilities (PP.H4) indicate the likelihood of a shared causal variant between gene expression and AD. Genes with PP.H4 > 0.8 were considered to have strong colocalization evidence.

**Figure 3:**
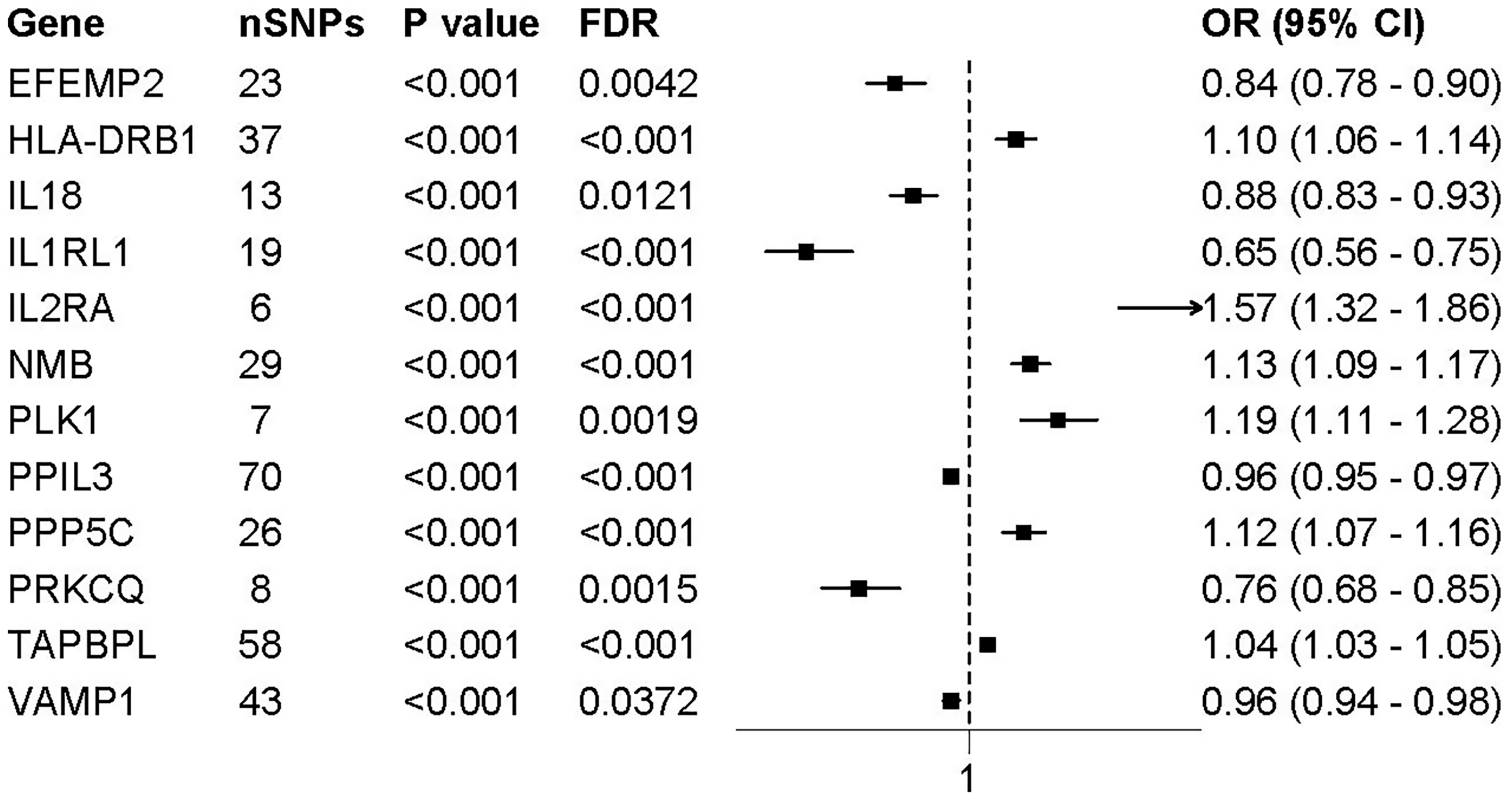
Forest plot of MR results for 12 colocalized druggable genes associated with AD. Forest plots presenting the odds ratios (ORs) and 95% confidence intervals (CIs) for the association between the expression levels of 12 colocalized druggable genes and AD, based on MR analysis. All genes shown passed colocalization (PP.H4 > 0.8) and multiple testing correction (FDR < 0.05) thresholds. Horizontal lines represent 95% CIs; squares indicate point estimates of ORs. Genes with OR > 1 suggest a positive association with AD risk, while OR < 1 indicates a protective effect.

We implemented colocalization analysis to assess the possibility of druggable genes and AD having a common causative variant (Supplementary Tables S5, S6). The findings suggested that AD and 12 vital genes likely shared a causative variant, with a posterior probability of PP.H4 exceeding 0.80 (Figures 2, 3). Consequently, the 12 significant genes were recognized as prospective therapeutic targets for AD.

#### 3.1.4 pQTL analysis

**Figure 4:**
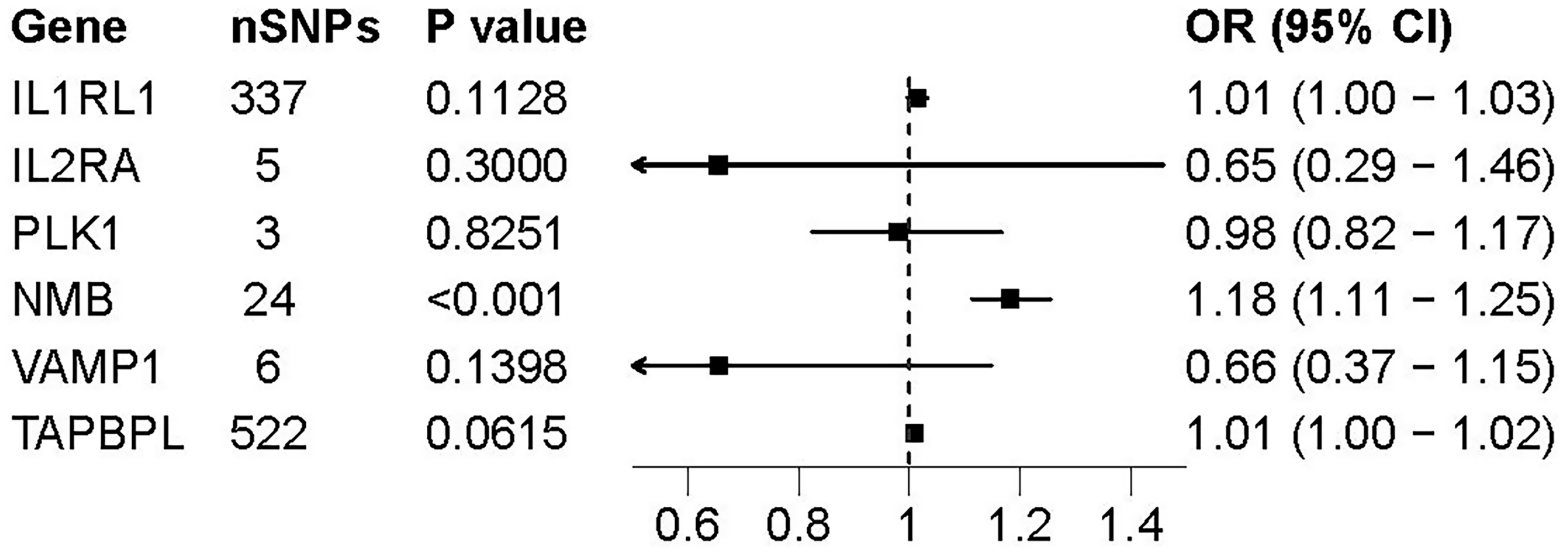
Forest plot of pQTL-based associations between 6 druggable genes and AD. Forest plots presenting the association between pQTL of six druggable genes and the risk of AD. ORs and 95% Cis are shown for each gene based on IVs estimates. Among the genes tested, only NMB showed a statistically significant association (*p* < 0.001), suggesting a potential causal role at the protein level.

The pQTL data for 6 potential drug targets were searched from deCODE genetics, while no data was found for the other 6 drug targets. In the process of selecting IVs representing drug targets protein level, we included the SNPs with *p* < 5.0 × 10^−8^ and clumped with *r*^2^ < 0.1 and clumping window of 10,000 kb. Nine hundred and two SNPs that fulfilled the criteria were selected as IVs for 7 potential drug targets (Supplementary Table S7). The MR result showed protein level of NMB in plasma was significantly associated with AD (OR = 1.18, *p* = 3.29 × 10^−8^) (Figure 4), MR-Egger regression more than 0.05 indicated no pleiotropy existed. The result was consistent with the findings of cis-eQTL analysis.

#### 3.1.5 SMR

In this study, SMR analysis using genetic variants that affect the expression of druggable gene as IVs was implemented to assess the causal relationships between NMB and AD (Supplementary Table S8). Using eQTL and *cis*-eQTL data for druggable genes as exposures, the SMR analysis revealed a significant association between plasma NMB levels and AD, with p_SMR values of 0.001451 and 0.000149, respectively (Table 1). The HEIDI test showed no pleiotropy in SMR analysis.

**Table 1:** NMB significantly associated with AD in summary-based Mendelian randomization (SMR) analysis.

Ensemble ID	CHR	Gene	Top SNP	SMR		HEIDI
				Beta	P-Value	P-Value
ENSG00000197696	15	NMB	rs2175567	0.199538	1.45E-03	8.64E-01
ENSG00000197696	15	NMB	rs1051168	0.128525	0.00014945	0.4022165

Summary data from SMR analysis evaluating the causal relationship between NMB expression and AD. “Top SNP” refers to the single nucleotide polymorphism most strongly associated with NMB expression. Beta indicates effect size; the HEIDI *p*-value tests for heterogeneity. A HEIDI *p* > 0.05 suggests no evidence of pleiotropy.

#### 3.1.6 NMB and other pruritic disease

**Figure 5:**
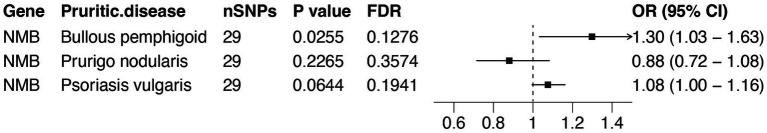
MR associations between NMB expression and multiple pruritic skin diseases. Forest plots presenting MR results for the association between NMB expression and the risk of three pruritic skin diseases: bullous pemphigoid (BP), prurigo nodularis, and psoriasis vulgaris. ORs and 95% CIs are shown. Among these, NMB was significantly associated with BP (OR = 1.30, 95% CI = 1.03–1.63, *p* = 0.0255), while associations with prurigo nodularis and psoriasis vulgaris did not reach statistical significance. These results suggest that NMB may play a broader role in pruritic skin diseases, particularly BP.

In order to estimate the causal relationships between NMB and other pruritic diseases including BP and psoriasis vulgaris. MR analysis was implemented again (Supplementary Table S9). The results demonstrated a significant association between NMB and bullous pemphigoid with a *p-*value of 0.0255. There is a probability that NMB may affect psoriasis vulgaris with *p*-value of 0.06; however, NMB did not influence prurigo nodularis (Figure 5).

#### 3.1.7 Drugs targeting NMB

Drugs targeting NMB were identified in the Drug SIGnatures database and are presented in Table 2. Metronidazole, 4-hydroxy-2-nonenal (HNE), thapsigargin, and progesterone downregulate NMB, while alsterpaullone, irinotecan, GW-8510, and camptothecin increase its expression.

**Table 2:** The regulatory effect of drugs targeting NMB.

Gene	Chemical name	Regulatory effect
NMB	Metronidazole	Decreases expression
NMB	Alsterpaullone	Increases expression
NMB	Irinotecan	Increases expression
NMB	GW-8510	Increases expression
NMB	Camptothecin	Increases expression
NMB	4-hydroxy-2-nonenal (HNE)	Decreases expression
NMB	Quercetin	Increases expression
NMB	67,526–95-8/thapsigargin	Decreases expression
NMB	Decitabine	Increases expression
NMB	Menadione	Increases expression
NMB	Benzo(a)pyrene	Increases expression
NMB	Aflatoxin B1	Affect expression
NMB	Progesterone	Decreases expression

Lists compounds identified from the Drug SIGnatures (DrugSig) database that modulate the expression of NMB. The table indicates whether each compound increases or decreases NMB expression. “Affect expression” denotes compounds reported to influence NMB expression without a clearly defined direction of regulation.

### 3.2 Validation of candidate genes expression in AD patient blood samples

**Figure 6:**
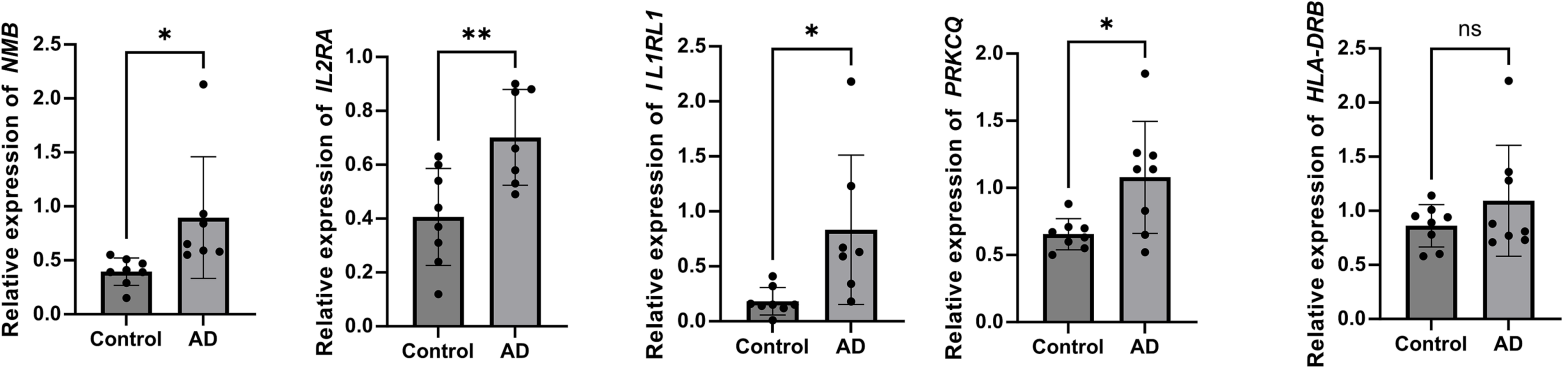
Relative expression of candidate genes in AD patients and healthy controls. Reverse transcription quantitative PCR (RT-qPCR) analysis showed significantly increased expression of NMB, IL2RA, IL1RL1, and PRKCQ in AD patients compared to controls. No significant difference was observed for HLA-DRB1. Expression levels were normalized to GAPDH and calculated using the 2^−ΔΔCt^ method. Mann–Whitney *U* test, **p < 0.01, *p < 0.05, and ns indicates no significant difference.

Quantitative PCR analysis was performed to validate the expression levels of five candidate genes (*NMB, IL2RA, IL1RL1, PRKCQ*, and *HLA-DRB1*) identified through colocalization analysis. Quantitative analysis revealed that the expression of *NMB, IL2RA, IL1RL1*, and *PRKCQ* was markedly elevated in individuals with atopic dermatitis relative to healthy controls (*p* < 0.05 or *p* < 0.01), indicating their possible involvement in disease development. Conversely, no significant expression change was observed for *HLA-DRB1* (*p* > 0.05), implying a limited or condition-specific role in AD pathology (Figure 6).

## 4 Discussion

Atopic dermatitis (AD) is a prevalent chronic inflammatory skin condition characterized by intense pruritus and an impaired skin barrier. The persistent “itch-scratch” cycle significantly impacts the physical development and mental health of affected individuals, particularly children, with an incidence rate of 2–10% (12). Alleviating pruritus remains the primary therapeutic goal for AD management. Neuro-epithelial-immune interactions are central to pruritus in AD, with notable infiltration of Th2 cells in lesional skin. Th2 cells secrete key cytokines, including IL-13, IL-31, and IL-4, which contribute to the development of pruritic symptoms (13). Although substantial progress has been made in developing cytokine-targeting therapies, a subset of patients shows limited response to current treatments, underscoring the need to identify novel drug targets (14).

In the present investigation, Mendelian randomization combined with colocalization methods was utilized to evaluate whether druggable gene expression is causally linked to atopic dermatitis (AD). The analysis highlighted Neuromedin B (NMB) as a promising therapeutic candidate. As part of the bombesin-like peptide family, NMB plays a key role in mediating itch signaling. Prior research indicates that spinal administration of NMB elicits scratching responses in a concentration-dependent fashion and facilitates the transmission of itch signals from dorsal root ganglia to the spinal cord (15, 16). Additionally, NMB interacts with other neuropeptides, such as gastrin-releasing peptide (GRP) and B-type natriuretic peptide (BNP), to modulate itch perception (17). While GRP primarily mediates non-histaminergic itch, NMB is strongly associated with histaminergic itch through its receptor, NMBR. Recent studies have revealed that NMB antagonists not only inhibit histaminergic itch but also significantly suppress chloroquine-induced non-histaminergic itch in murine models, indicating a broader role for NMB in pruritus regulation (18).

Studies have highlighted the involvement of NMB in AD-associated pruritus, focusing on its expression levels and interactions with other neuropeptides. NMB-mediated scratching behavior could be enhanced by BNP, the possible mechanism is that NMB activates PLCβ signaling at a low concentration, while BNP stimulates Gai signaling, subsequently activating PLCβ to transmit itch sensation (17). Cross-inhibition between NMB and GRP in the spinal cord further underscores the complexity of neuropeptide-mediated itch signaling in AD. Consistent with these findings, microarray-based transcriptomic studies have reported upregulated NMB expression in AD skin models (19).

Beyond its role in pruritus, NMB may regulate type 2 immune responses by modulating group 2 innate lymphoid cells (ILC2s), key mediators of neuro-epithelial-immune crosstalk. ILC2s respond to epithelial-derived cytokines and neural signals to maintain skin homeostasis (20). Notably, NMB has been reported to attenuate ILC2 responses by increasing NMB receptor expression on ILC2s, thereby reducing the production of IL-13 and IL-5 (21).

A number of pharmacological agents acting on NMB-related pathways have been reported to exert beneficial effects in the context of atopic dermatitis. Among them, quercetin—a plant-derived flavonoid recognized for its strong anti-inflammatory and immune-regulatory capacity—has been effective in reducing AD-associated inflammation. This is achieved through the inhibition of pro-inflammatory mediators including TNF-*α*, IL-6, and IFN-*γ*, along with attenuation of the NF-κB signaling pathway (22, 23). Additionally, quercetin modulates Th1/Th2 immune responses, reduces serum IgE levels, and inhibits histamine H4 receptor-mediated calcium influx to alleviate pruritus (24). Although quercetin is widely reported to suppress systemic inflammation, concentration-dependent neuro-modulatory effects have been described. Low concentrations (10–20 μM) protect neurons via antioxidant and anti-inflammatory actions, whereas high concentrations (> 50 μM) disrupt mitochondrial membranes and elicit pro-oxidant activity that can amplify neurogenic inflammation (25, 26). Likewise, chronic high-dose administration *in vivo* (> 50 mg kg^−1^ day^−1^) triggers apoptosis and other adverse effects (25). Such bidirectional, concentration-dependent effects reconcile our in-vitro observations with the epidemiological benefits reported for dietary quercetin.

Metronidazole has demonstrated the ability to reduce inflammation, suppress immune overactivation, and alleviate pruritus in experimental settings involving inflammatory skin conditions (27). Furthermore, elevated progesterone levels during pregnancy have been associated with increased Th2 activity via Jak1 pathway activation, suggesting a complex interplay between hormonal regulation and AD pathogenesis (28, 29). Research on NMB antagonists has advanced actively in recent years. Several small molecules (e.g., PD168368, PD165929) and peptidic ligands (e.g., BIM-23042) have been shown to bind NMBR with high affinity, thereby blocking downstream Ca^2+^, MAPK and PI3K signaling (30, 31). In pain models, NMBR antagonists significantly attenuate neurogenic inflammation (32). Although in vivo validation in AD model is still lacking, the pivotal role of NMB/NMBR signaling in neuroinflammation and synaptic plasticity positions the development of highly selective NMB antagonists as a promising new therapeutic strategy for AD.

Pollutant-induced oxidative stress is another potential modulator of NMB pathways in AD. Elevated levels of HNE, a lipid peroxidation product, have been implicated in exacerbating inflammatory skin conditions (33). HNE activates transient receptor potential ankyrin 1 (TRPA1) channel, promoting inflammation in murine model (33). Similarly, thapsigargin, an ATPase inhibitor, promotes basophil degranulation by depleting intracellular calcium stores. Given the potential of HNE and thapsigargin to modulate NMB pathways and exacerbate AD, further investigation is warranted.

Subsequent MR analysis indicated that NMB may also play a role in other pruritic dermatological disorders. Both BP and PN are classified as Th2-dominant pruritic diseases, BP—but not PN—exhibits a neuro-sensitizing immune signature (high IL-31, IgE-autoantibodies, and eosinophil-derived neurotoxins) that aligns with the NMB-associated pruritus phenotype (34). Conversely, psoriasis-associated pruritus involves IL-17-driven neuronal activation and IL-31/OSM signaling, partially overlapping with the BP signature (35). These data suggest that NMB association reflects specific neuro-immune convergence rather than canonical T-helper polarity. In BP, pruritus plays a major role in diminishing patient comfort and quality of life. This symptom is largely attributed to autoantibodies directed against the basement membrane, which in turn stimulate mast cells and promote the secretion of itch-inducing factors, including histamine and IL-31 (36). The specific role of NMB in BP including its potential interaction with mast cells, cytokines, and neural pathways involved in pruritus, warrants further investigation.

To further support the MR findings, we performed RT-qPCR validation of candidate genes in peripheral blood samples from AD patients. Notably, *NMB, IL2RA, IL1RL1*, and *PRKCQ* showed significantly increased expression in AD patients, providing biological support for their involvement in AD pathogenesis. This experimental validation aligns with the MR and colocalization results, reinforcing the reliability of our genetic findings. However, *HLA-DRB1* did not show a significant expression difference, possibly due to tissue-specific regulation or limited sample size. Interestingly, *IL1RL1* and *PRKCQ* were predicted to be protective in MR analysis (OR < 1), yet showed increased expression in AD patients. This discrepancy may reflect the difference between genetically predicted expression and actual expression under disease conditions, where inflammation-driven regulatory mechanisms may override baseline genetic effects. These observations highlight the importance of integrating genetic inference with molecular validation and call for further studies in lesional skin and larger cohorts.

This study presents several strengths. First, the use of MR analysis, combined with colocalization and pQTL analyses, enhances the robustness of our findings. Second, we selected candidate genes for experimental validation based on strict statistical criteria, including MR significance, colocalization probability, and effect size, ensuring that only the most promising targets were prioritized for follow-up. Third, we performed RT-qPCR validation in peripheral blood samples from AD patients, which confirmed the upregulation of *NMB, IL2RA, IL1RL1*, and *PRKCQ*, further supporting the MR-based predictions.

Nonetheless, it is important to recognize certain limitations. The pQTL data were derived from a cohort of Icelandic individuals, whereas the GWAS data for AD predominantly originated from other European populations. This ancestry discrepancy may introduce population structure bias. Future work could exploit pan-European pQTL meta-analyses and trans-ethnic MR-MEGA to further dilute any residual population structure bias. In addition, our validation was performed in peripheral blood samples, which may not fully reflect expression patterns in lesional skin. The limited number of participants in the PCR analysis, along with the non-significant results observed for *HLA-DRB1*, suggest that further validation in larger cohorts and disease-relevant tissues is warranted. Finally, while genetic evidence provides strong support for target prioritization, clinical trials remain essential to confirm real-world therapeutic potential.

In conclusion, we employed MR to identify potential druggable targets for AD, with NMB emerging as a key candidate supported by colocalization and pQTL analyses. RT-qPCR validation confirmed significantly increased expression of *NMB, IL2RA, IL1RL1,* and *PRKCQ* in AD patients, reinforcing their potential relevance to disease pathogenesis. Among them, NMB stands out for its dual role in itch signaling and immune regulation, making it a particularly promising therapeutic target. Moreover, its potential involvement in bullous pemphigoid suggests broader clinical implications. These results offer valuable perspectives for the development of novel treatments for AD and related pruritic disorders.

## Data Availability

The datasets presented in this study can be found in online repositories. The names of the repository/repositories and accession number(s) can be found in the article/Supplementary material.

## Footnotes

^1^ https://eqtlgen.org/

^2^ https://www.decode.com/publications/

^3^ https://www.finngen.fi/en
