# Supplementary material for: Neuromedin B identified as a therapeutic target for atopic dermatitis: evidence from Mendelian randomization and PCR validation
Source: Front Med (Lausanne). 2025 Nov 19;12:1660249. doi: 10.3389/fmed.2025.1660249 (PMC12672242; doi:10.3389/fmed.2025.1660249)
Supplement: Supplementary file 1 [file Data_Sheet_1.zip › Supplementary Material/MR leave−one−out sensitivity analysis .pdf]

MR leave–one–out sensitivity analysis for 'exposure' on 'outcome'

ADCK3

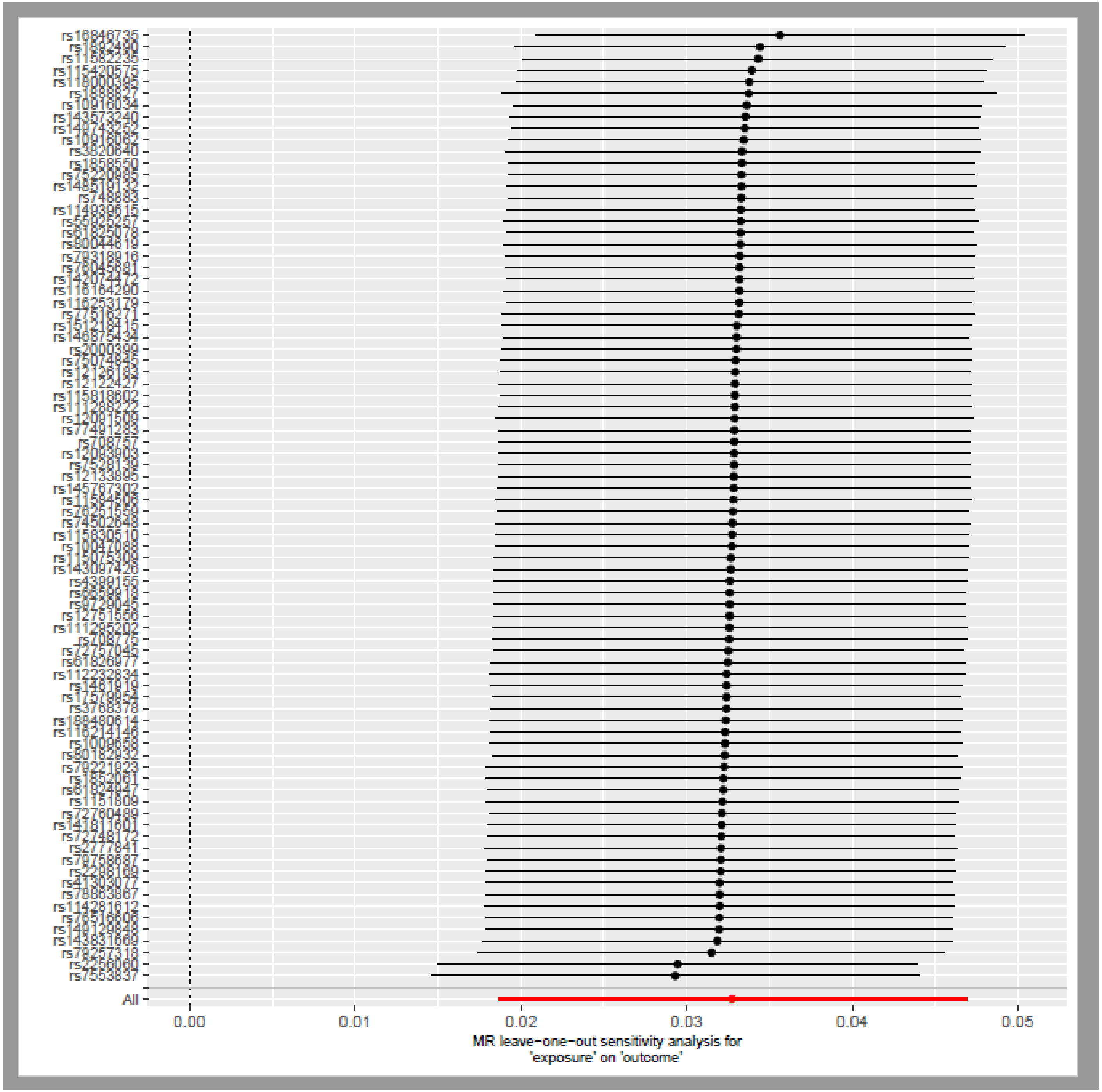
All
0.00
0.01
0.02
0.03
0.04
0.05
MR leave-one-out sensitivity analysis for
'exposure' on 'outcome'

BLK

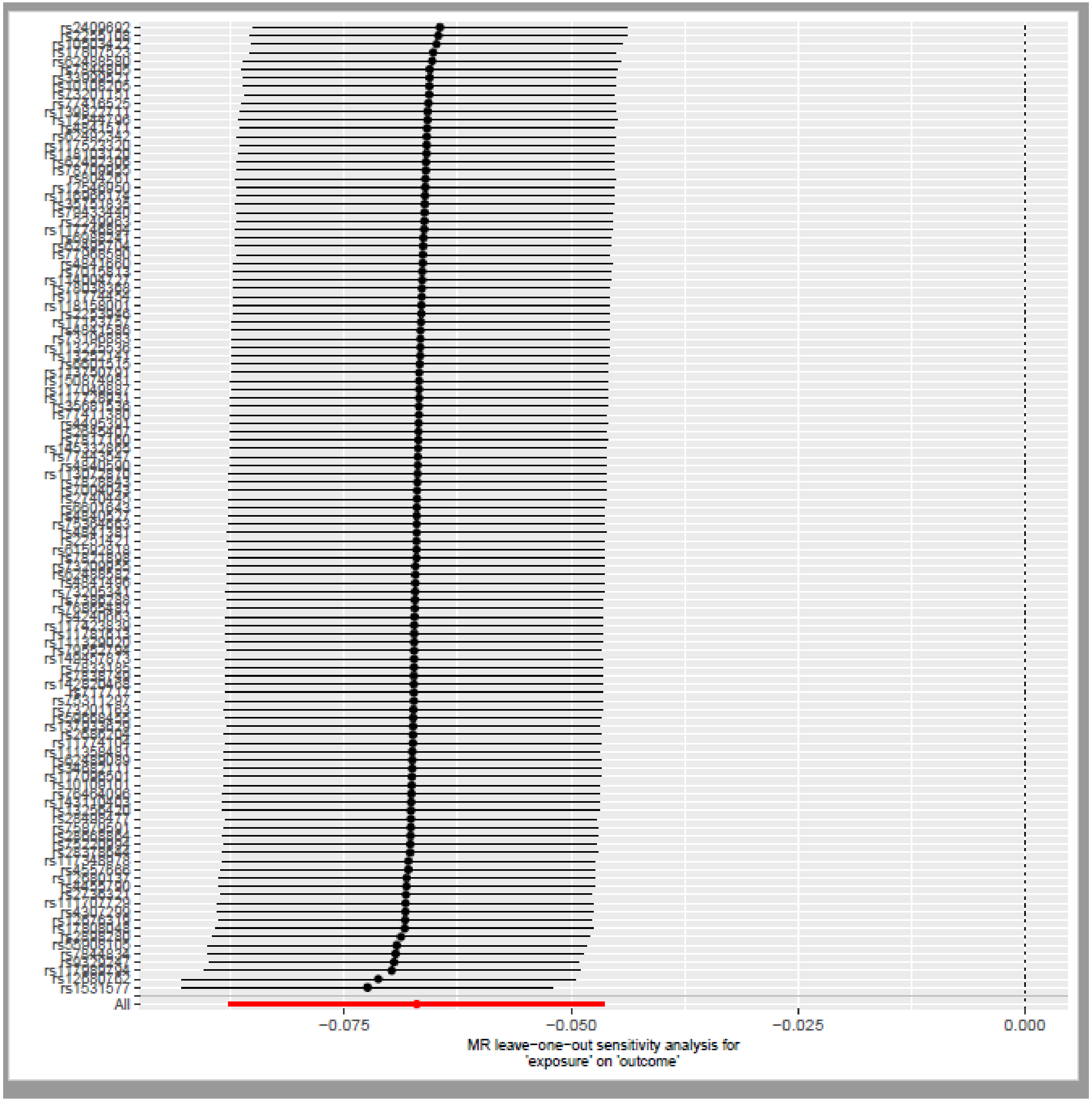
All
−0.075
−0.050
−0.025
0.000
MR leave−one−out sensitivity analysis for
'exposure' on 'outcome'

CCR2

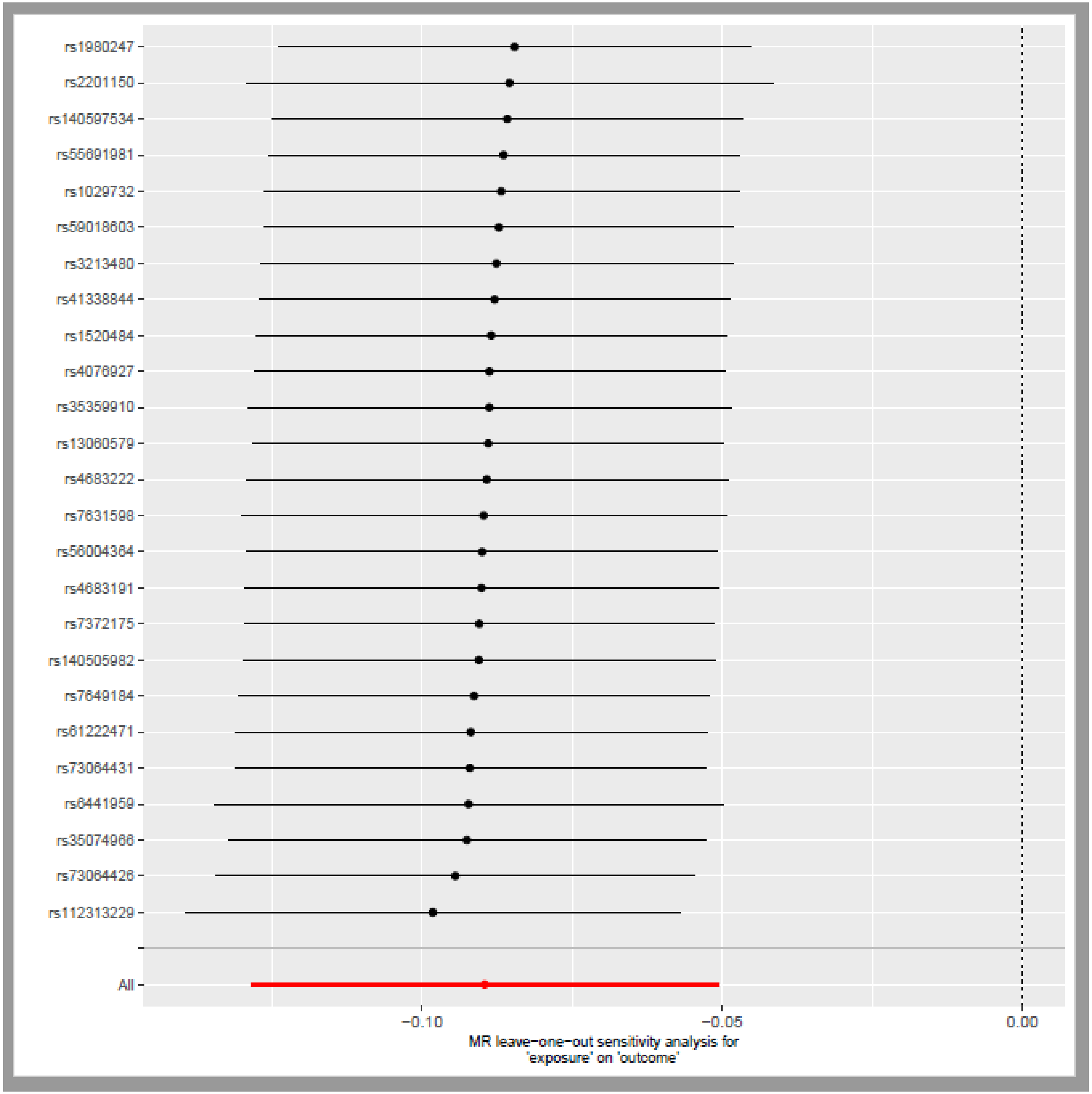
rs1980247
rs2201150
rs140597534
rs55691981
rs1029732
rs59018603
rs3213480
rs41338844
rs1520484
rs4076927
rs35359910
rs13060579
rs4683222
rs7631598
rs56004364
rs4683191
rs7372175
rs140505982
rs7649184
rs61222471
rs73064431
rs6441959
rs35074966
rs73064426
rs112313229
All
−0.10
−0.05
0.00
MR leave−one−out sensitivity analysis for
'exposure' on 'outcome'

CD247

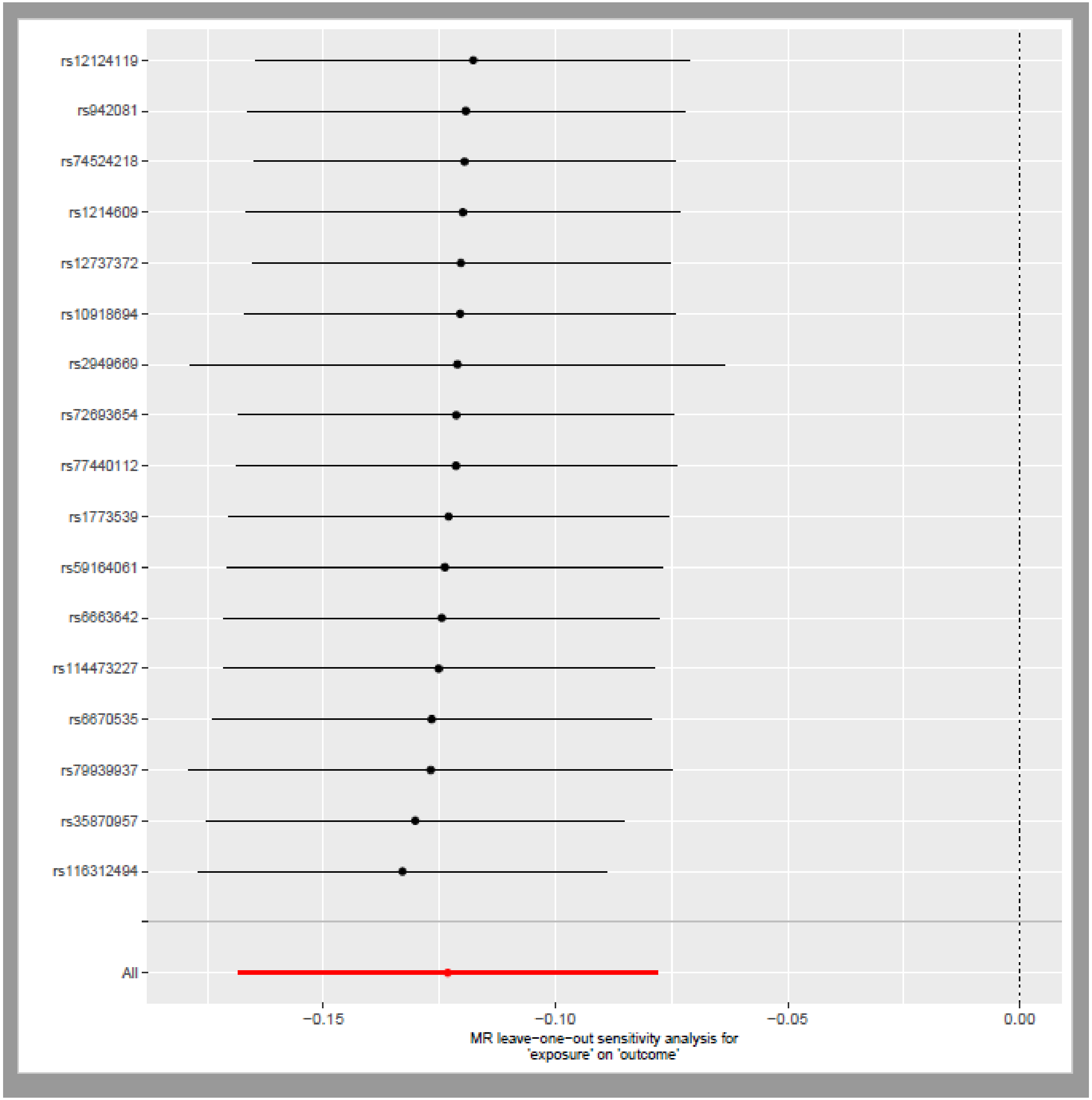
rs12124119
rs942081
rs74524218
rs1214609
rs12737372
rs10918694
rs2949669
rs72693654
rs77440112
rs1773539
rs59164061
rs6663642
rs114473227
rs6670535
rs79939937
rs35870957
rs116312494
All
−0.15
−0.10
−0.05
0.00
MR leave−one−out sensitivity analysis for
'exposure' on 'outcome'

CDR5R1

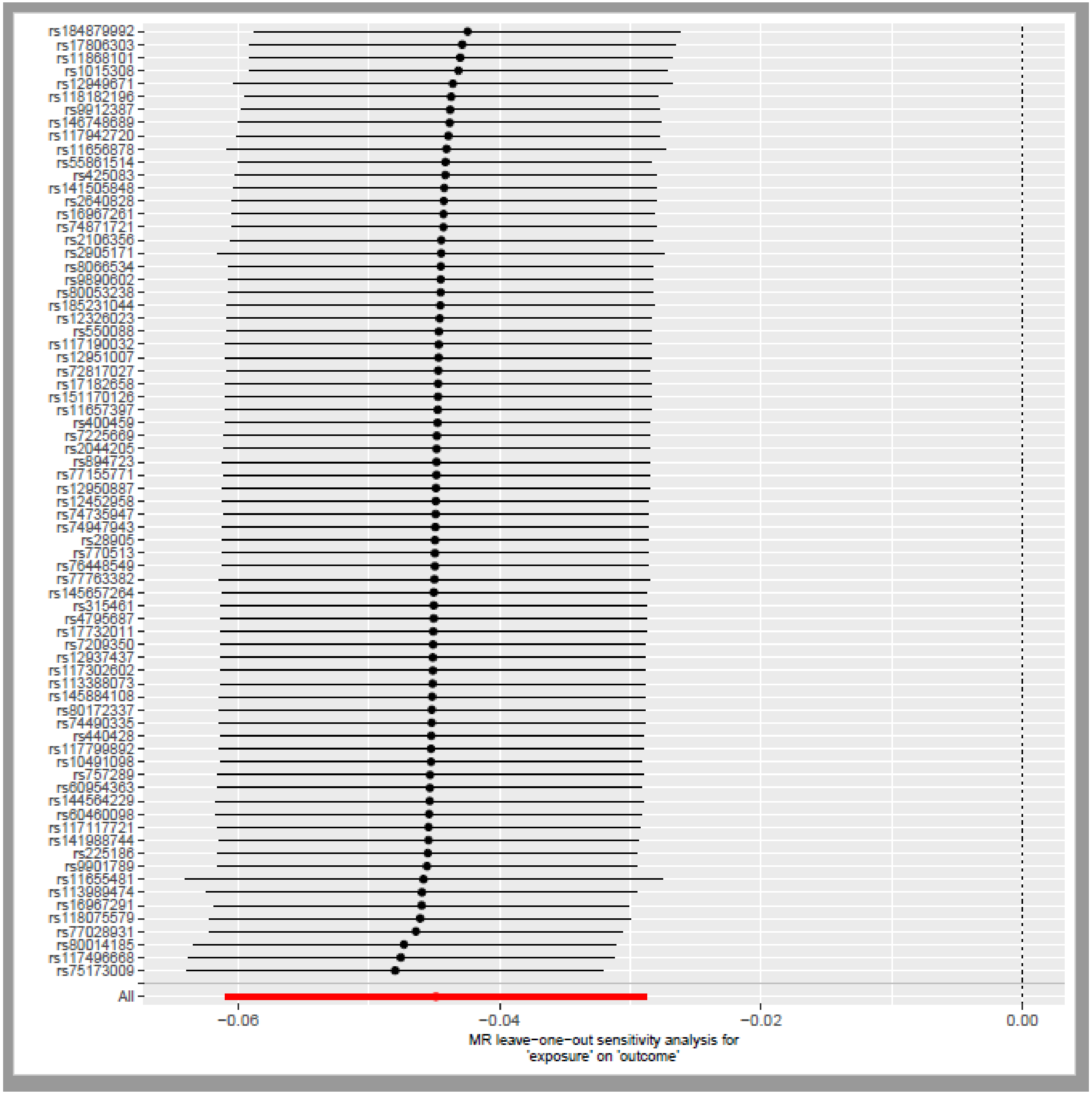

rs184879992
rs17806303
rs11868101
rs1015308
rs12949671
rs118182196
rs9912387
rs146748689
rs117942720
rs11656878
rs55861514
rs425083
rs141505848
rs2640828
rs16967261
rs74871721
rs2106356
rs2905171
rs8066534
rs9890602
rs80053238
rs185231044
rs12326023
rs550088
rs117190032
rs12951007
rs72817027
rs17182658
rs151170126
rs11657397
rs400459
rs7225669
rs2044205
rs894723
rs77155771
rs12950887
rs12452958
rs74735947
rs74947943
rs28905
rs770513
rs76448549
rs77763382
rs145657264
rs315461
rs4795687
rs17732011
rs7209350
rs12937437
rs117302602
rs113388073
rs145884108
rs80172337
rs74490335
rs440428
rs117799892
rs10491098
rs757289
rs60954363
rs144564229
rs60460098
rs117117721
rs141988744
rs225186
rs9901789
rs11655481
rs113989474
rs16967291
rs118075579
rs77028931
rs80014185
rs117496668
rs75173009
All
−0.06
−0.04
−0.02
0.00
MR leave−one−out sensitivity analysis for
'exposure' on 'outcome'

CORIN

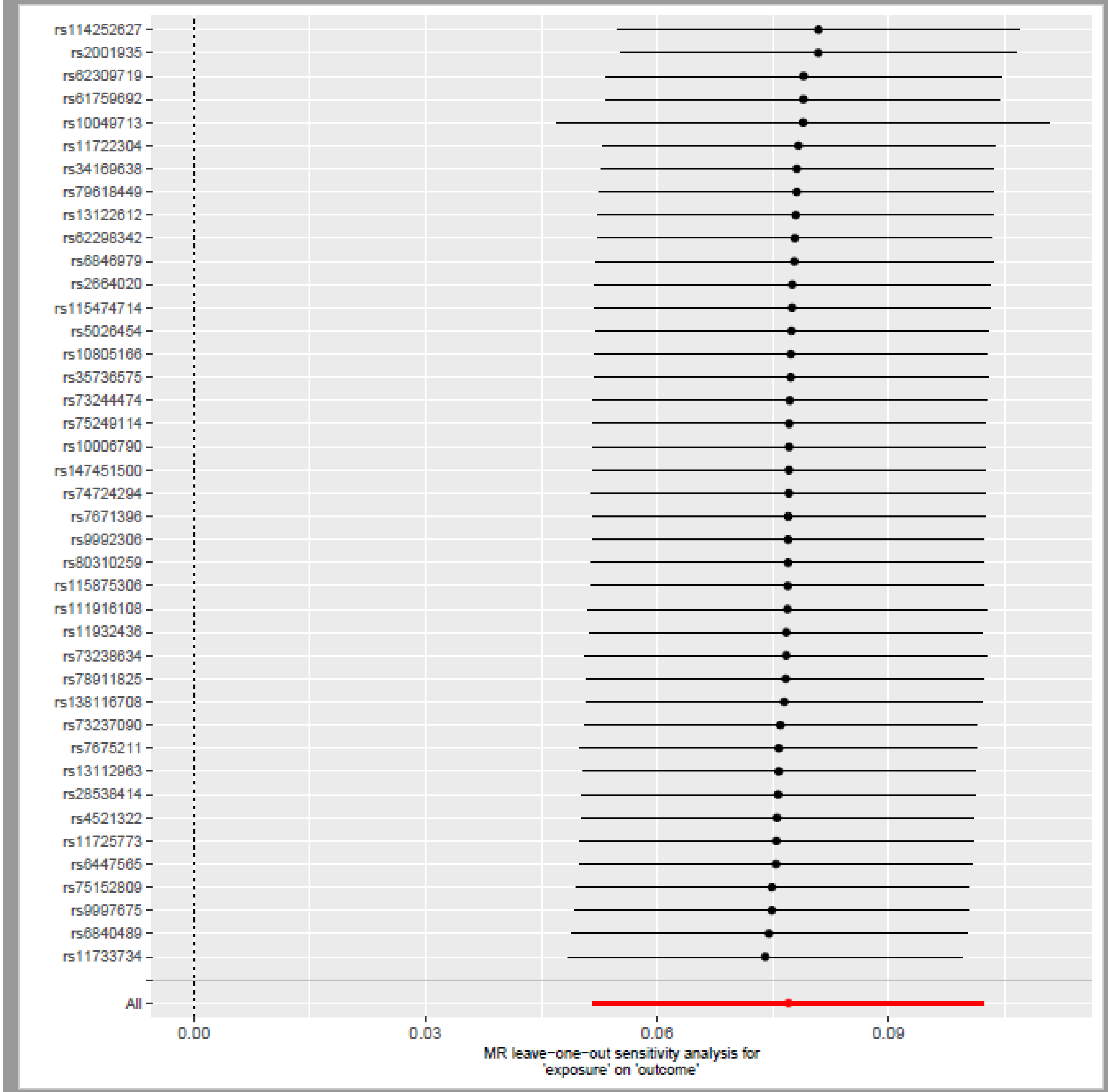
rs114252627
rs2001935
rs62309719
rs61759692
rs10049713
rs11722304
rs34169638
rs79618449
rs13122612
rs62298342
rs6846979
rs2664020
rs115474714
rs5026454
rs10805166
rs35736575
rs73244474
rs75249114
rs10006790
rs147451500
rs74724294
rs7671396
rs9992306
rs80310259
rs115875306
rs111916108
rs11932436
rs73238634
rs78911825
rs138116708
rs73237090
rs7675211
rs13112963
rs28538414
rs4521322
rs11725773
rs6447565
rs75152809
rs9997675
rs6840489
rs11733734
All
0.00
0.03
0.06
0.09
MR leave−one−out sensitivity analysis for
'exposure' on 'outcome'

EFEMP2

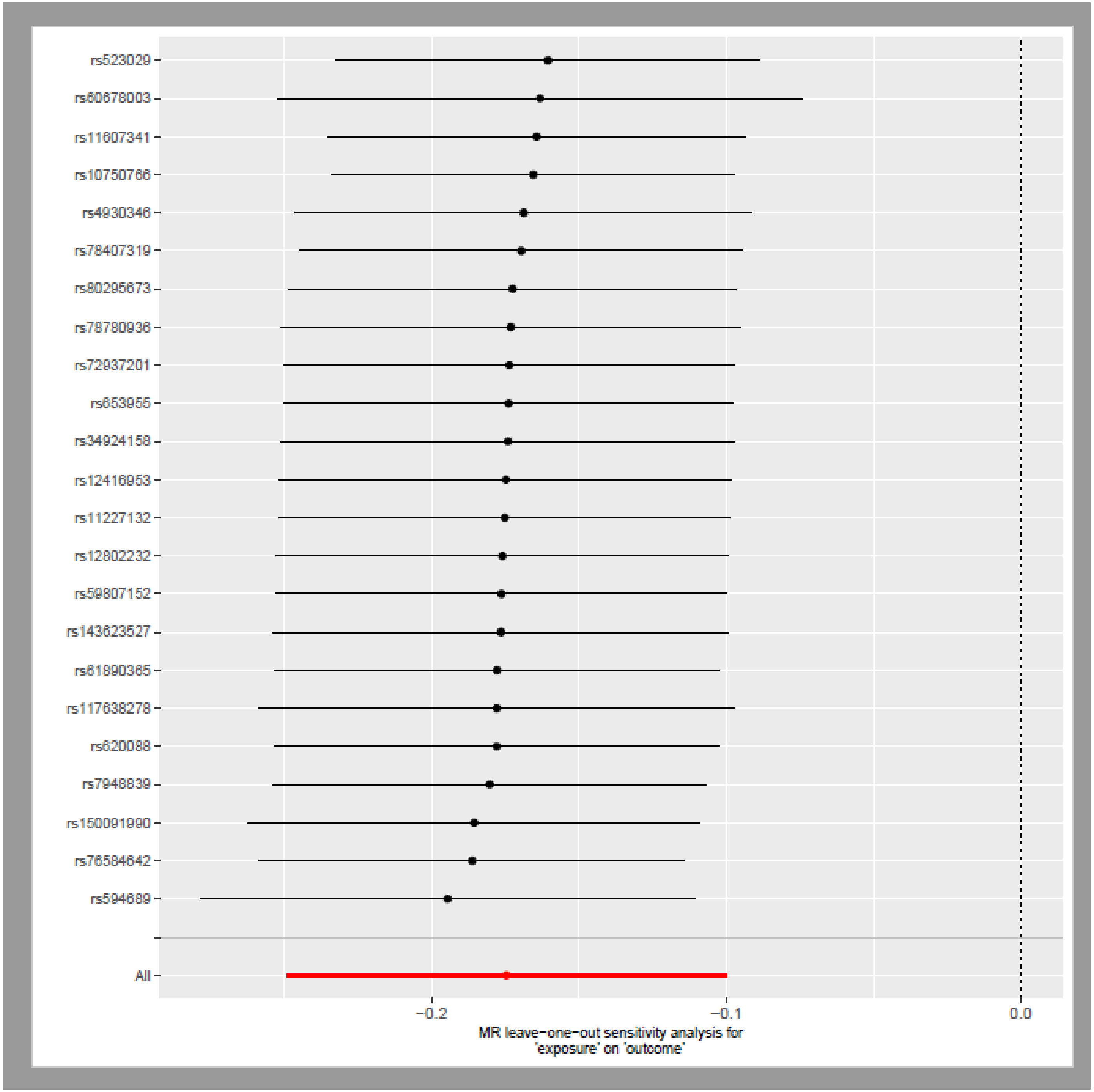

rs523029
rs60678003
rs11607341
rs10750766
rs4930346
rs78407319
rs80295673
rs78780936
rs72937201
rs653955
rs34924158
rs12416953
rs11227132
rs12802232
rs59807152
rs143623527
rs61890365
rs117638278
rs620088
rs7948839
rs150091990
rs76584642
rs594689
All
−0.2
−0.1
0.0
MR leave−one−out sensitivity analysis for 'exposure' on 'outcome'

GPX3

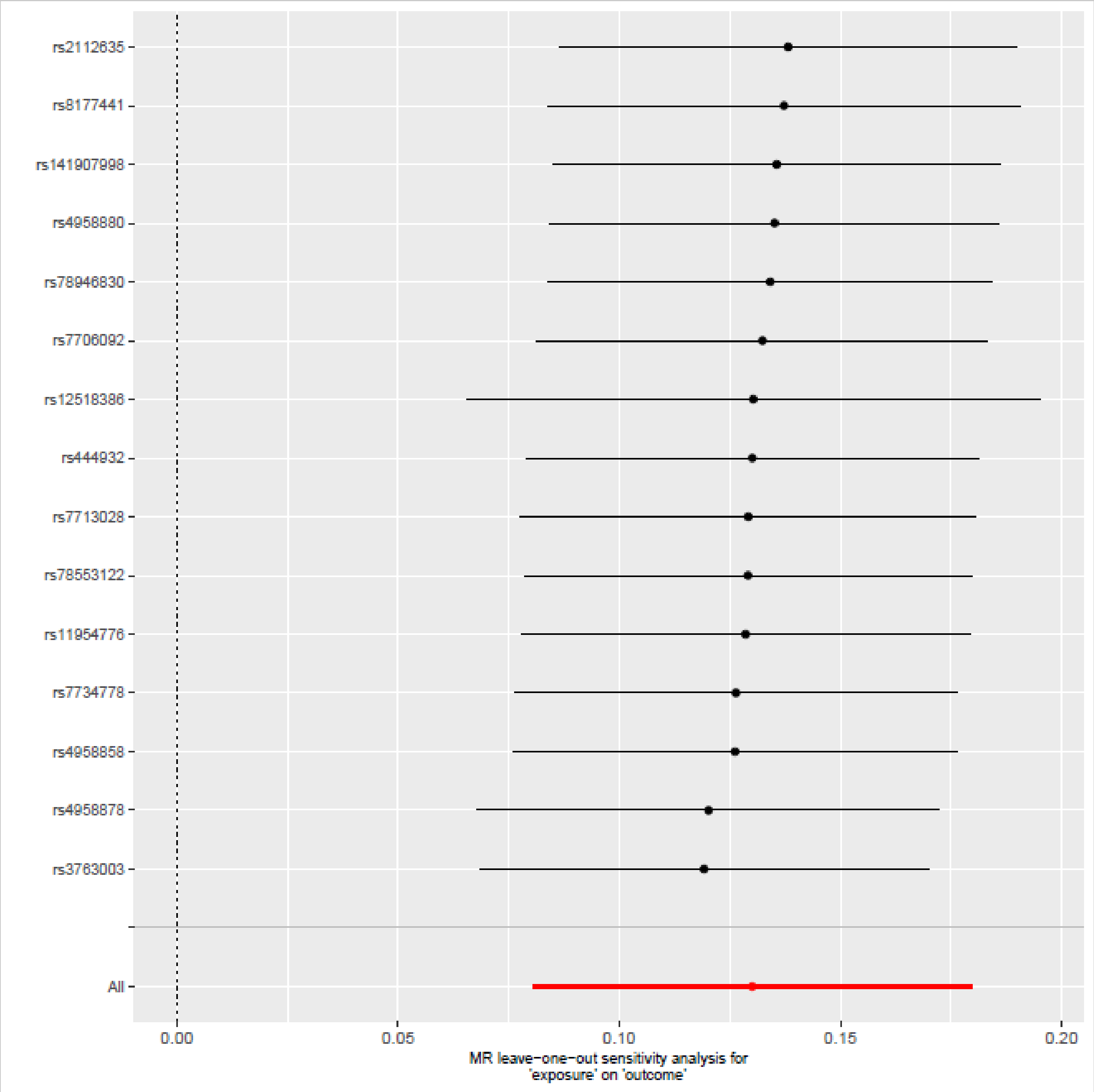

rs2112635
rs8177441
rs141907998
rs4958880
rs78946830
rs7706092
rs12518386
rs444932
rs7713028
rs78553122
rs11954776
rs7734778
rs4958858
rs4958878
rs3763003
All
0.00
0.05
0.10
0.15
0.20
MR leave−one−out sensitivity analysis for
'exposure' on 'outcome'

HLA-DRB1

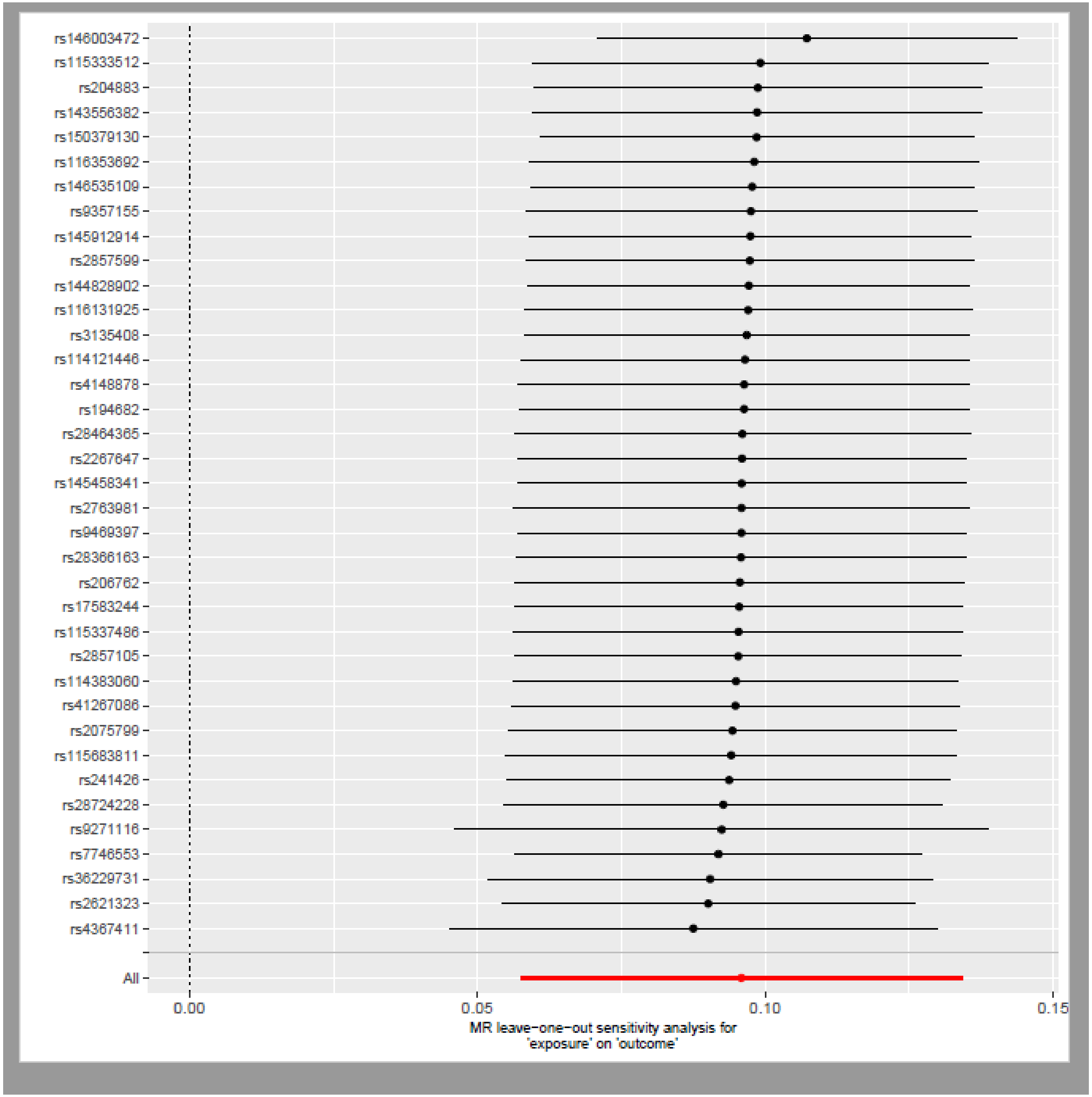
rs146003472
rs115333512
rs204883
rs143556382
rs150379130
rs116353692
rs146535109
rs9357155
rs145912914
rs2857599
rs144828902
rs116131925
rs3135408
rs114121446
rs4148878
rs194682
rs28464365
rs2267647
rs145458341
rs2763981
rs9469397
rs28366163
rs206762
rs17583244
rs115337486
rs2857105
rs114383060
rs41267086
rs2075799
rs115683811
rs241426
rs28724228
rs9271116
rs7746553
rs36229731
rs2621323
rs4367411
All
0.00
0.05
0.10
0.15
MR leave-one-out sensitivity analysis for
'exposure' on 'outcome'

IL 18

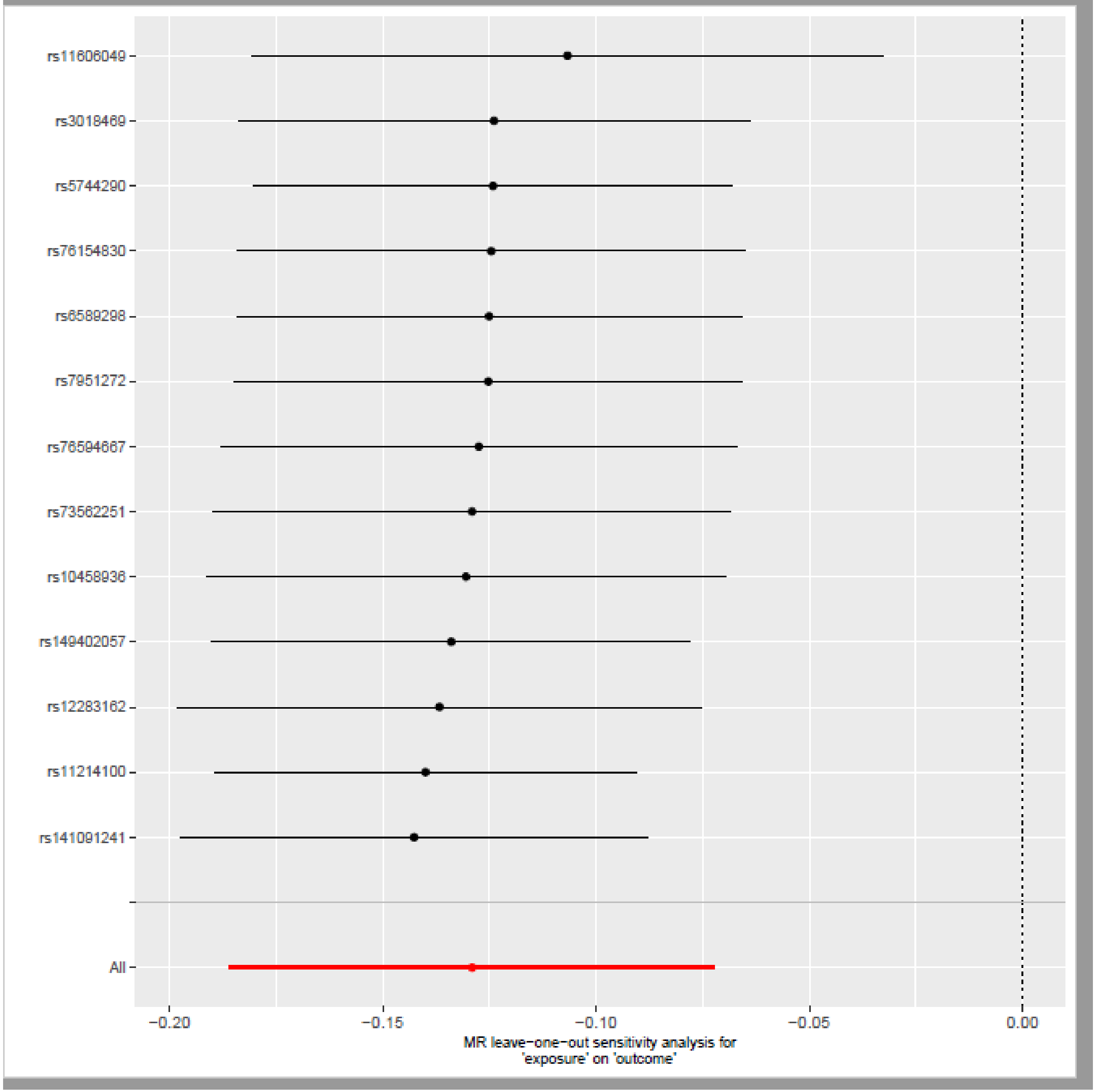
rs11606049
rs3018469
rs5744290
rs76154830
rs6589298
rs7951272
rs76594667
rs73562251
rs10458936
rs149402057
rs12283162
rs11214100
rs141091241
All
−0.20
−0.15
−0.10
−0.05
0.00
MR leave−one−out sensitivity analysis for
'exposure' on 'outcome'

IL1RL1

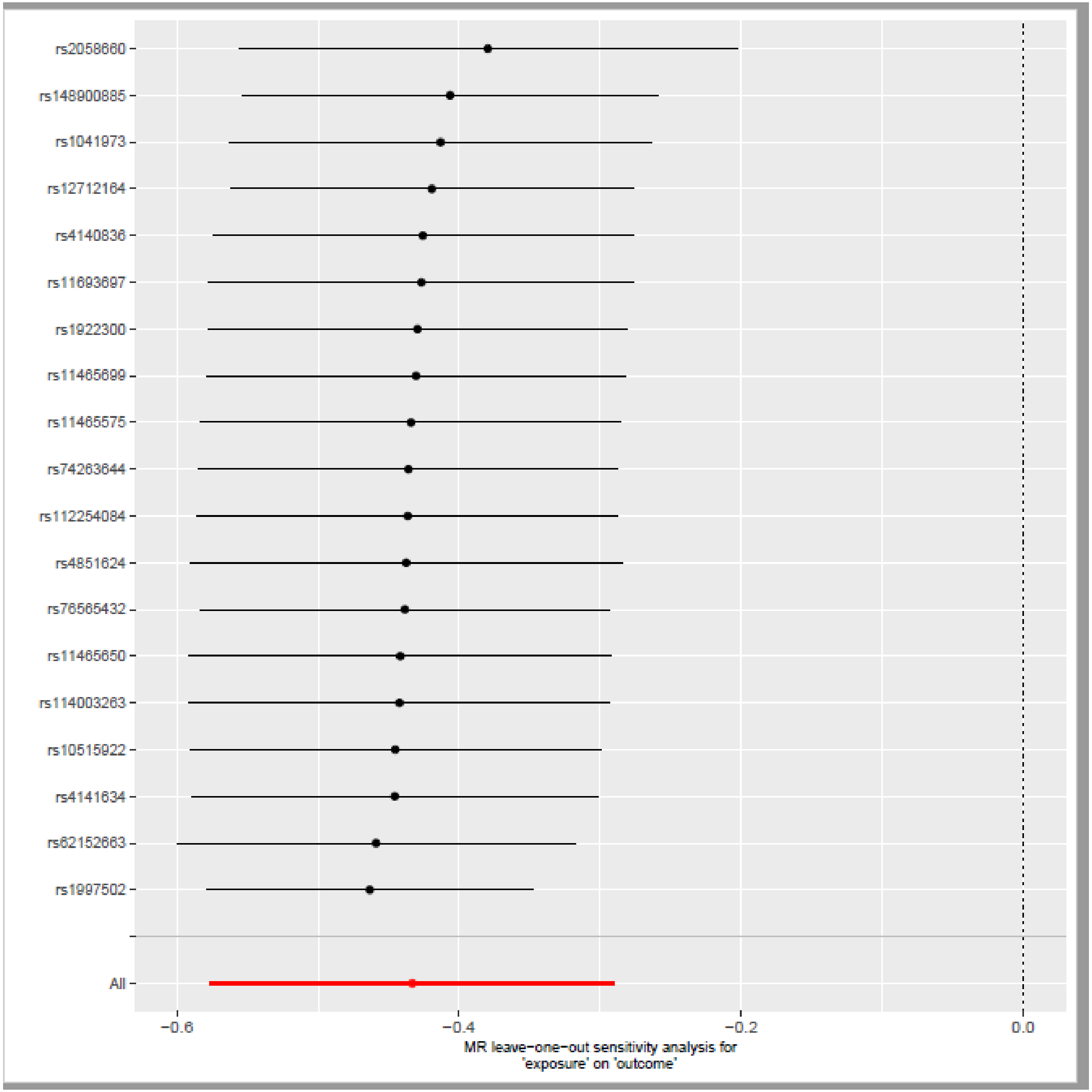
rs2058660
rs148900885
rs1041973
rs12712164
rs4140836
rs11693697
rs1922300
rs11465699
rs11465575
rs74263644
rs112254084
rs4851624
rs76565432
rs11465650
rs114003263
rs10515922
rs4141634
rs62152663
rs1997502
All
−0.6
−0.4
−0.2
0.0
MR leave−one−out sensitivity analysis for
'exposure' on 'outcome'

IL2RA

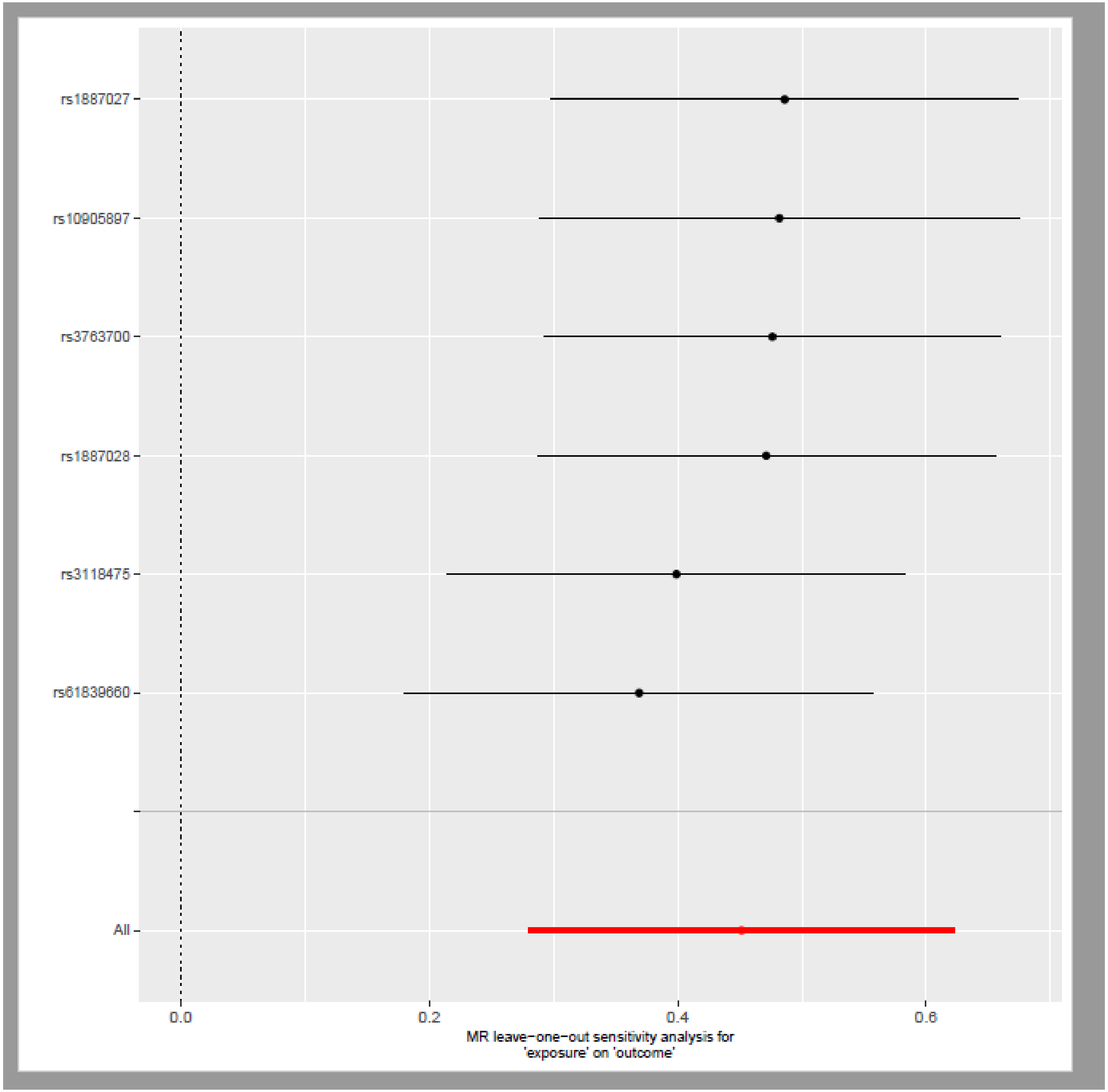
rs1887027
rs10905897
rs3763700
rs1887028
rs3118475
rs61839660
All
0.0
0.2
0.4
0.6
MR leave−one−out sensitivity analysis for
'exposure' on 'outcome'

ITM2B

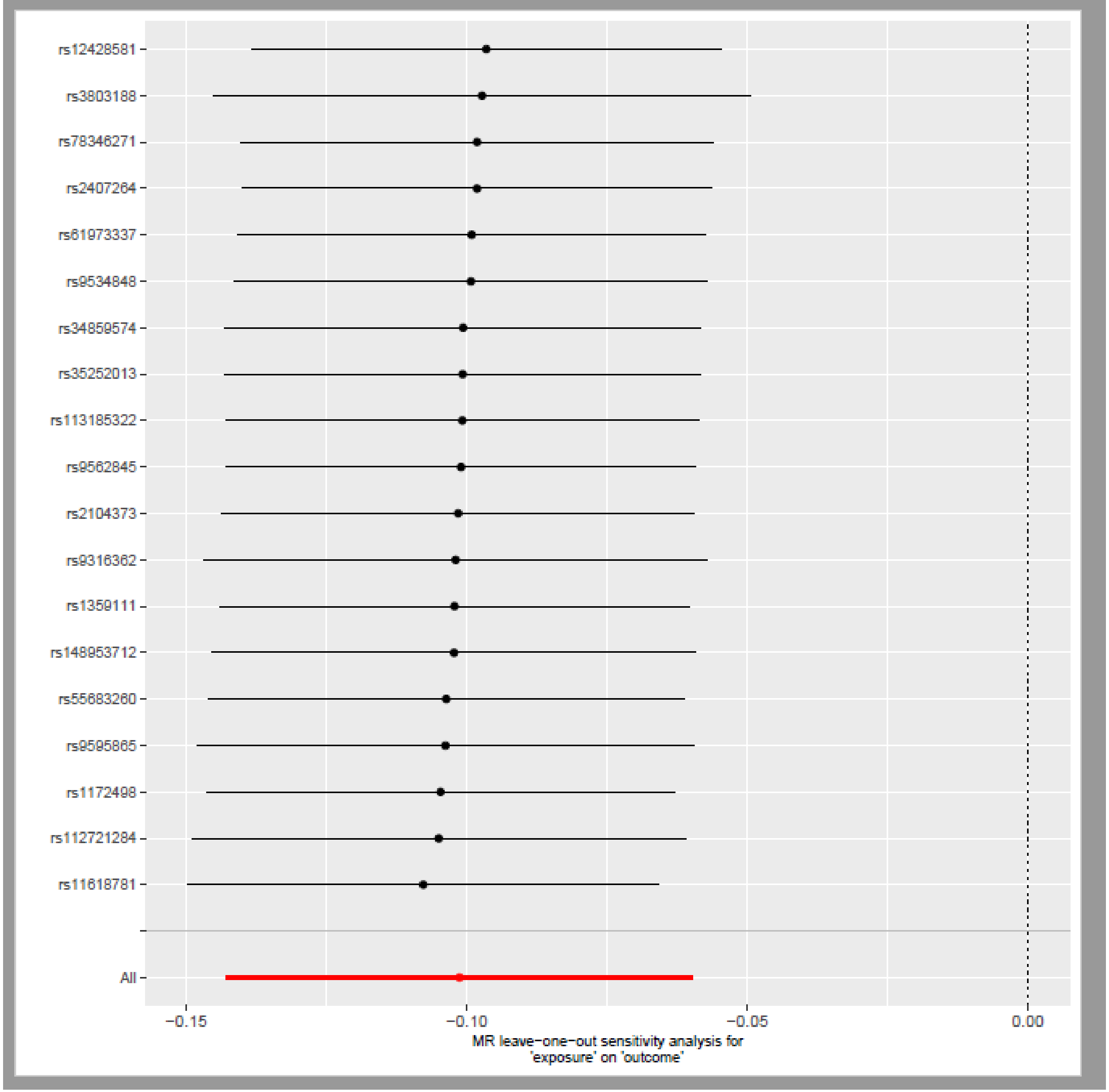
rs12428581
rs3803188
rs78346271
rs2407264
rs61973337
rs9534848
rs34859574
rs35252013
rs113185322
rs9562845
rs2104373
rs9316362
rs1359111
rs148953712
rs55683260
rs9595865
rs1172498
rs112721284
rs11618781
All
−0.15
−0.10
−0.05
0.00
MR leave−one−out sensitivity analysis for
'exposure' on 'outcome'

ITPR3

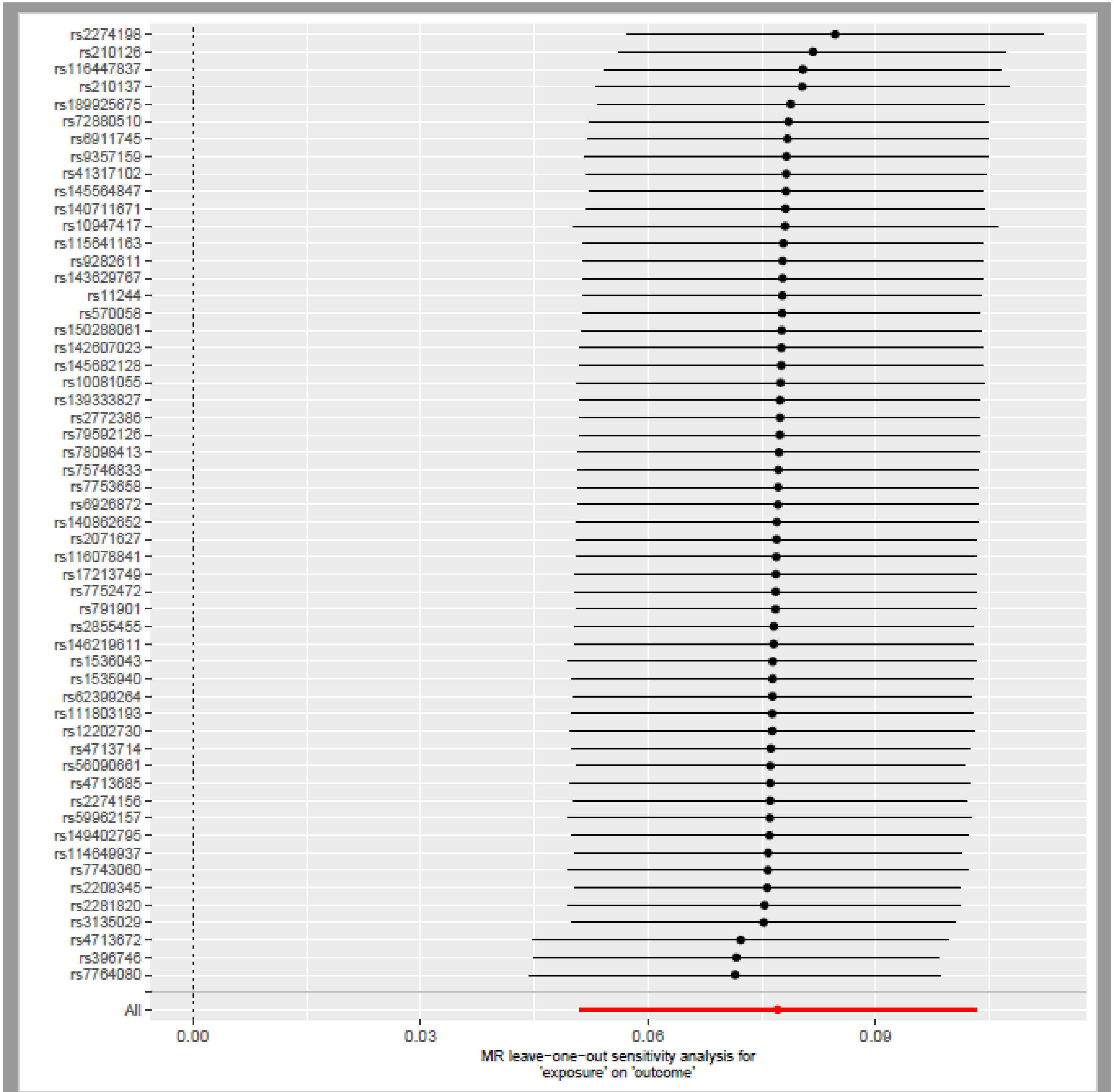
rs2274198
rs210126
rs116447837
rs210137
rs189925675
rs72880510
rs6911745
rs9357159
rs41317102
rs145564847
rs140711671
rs10947417
rs115641163
rs9282611
rs143629767
rs11244
rs570058
rs150288061
rs142607023
rs145682128
rs10081055
rs139333827
rs2772386
rs79592126
rs78098413
rs75746833
rs7753658
rs6926872
rs140862652
rs2071627
rs116078841
rs17213749
rs7752472
rs791901
rs2855455
rs146219611
rs1536043
rs1535940
rs62399264
rs111803193
rs12202730
rs4713714
rs56090661
rs4713685
rs2274156
rs59962157
rs149402795
rs114649937
rs7743060
rs2209345
rs2281820
rs3135029
rs4713672
rs396746
rs7764080
All
0.00
0.03
0.06
0.09
MR leave−one−out sensitivity analysis for
'exposure' on 'outcome'

MAPK3

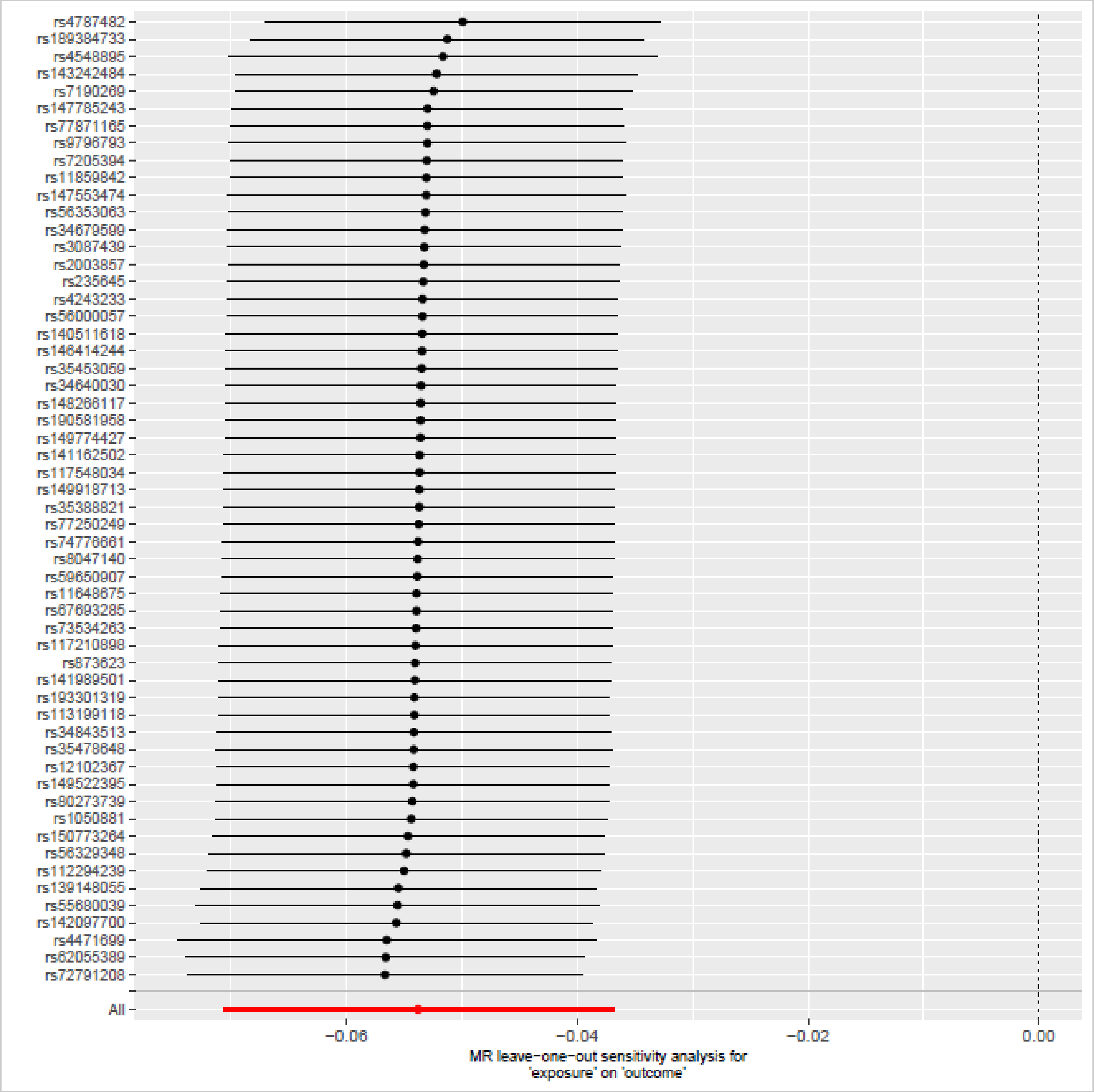
rs4787482
rs189384733
rs4548895
rs143242484
rs7190269
rs147785243
rs77871165
rs9796793
rs7205394
rs11859842
rs147553474
rs56353063
rs34679599
rs3087439
rs2003857
rs235645
rs4243233
rs56000057
rs140511618
rs146414244
rs35453059
rs34640030
rs148266117
rs190581958
rs149774427
rs141162502
rs117548034
rs149918713
rs35388821
rs77250249
rs74776661
rs8047140
rs59650907
rs11648675
rs67693285
rs73534263
rs117210898
rs873623
rs141989501
rs193301319
rs113199118
rs34843513
rs35478648
rs12102367
rs149522395
rs80273739
rs1050881
rs150773264
rs56329348
rs112294239
rs139148055
rs55680039
rs142097700
rs4471699
rs62055389
rs72791208
All
−0.06
−0.04
−0.02
0.00
MR leave−one−out sensitivity analysis for
'exposure' on 'outcome'

NCR3

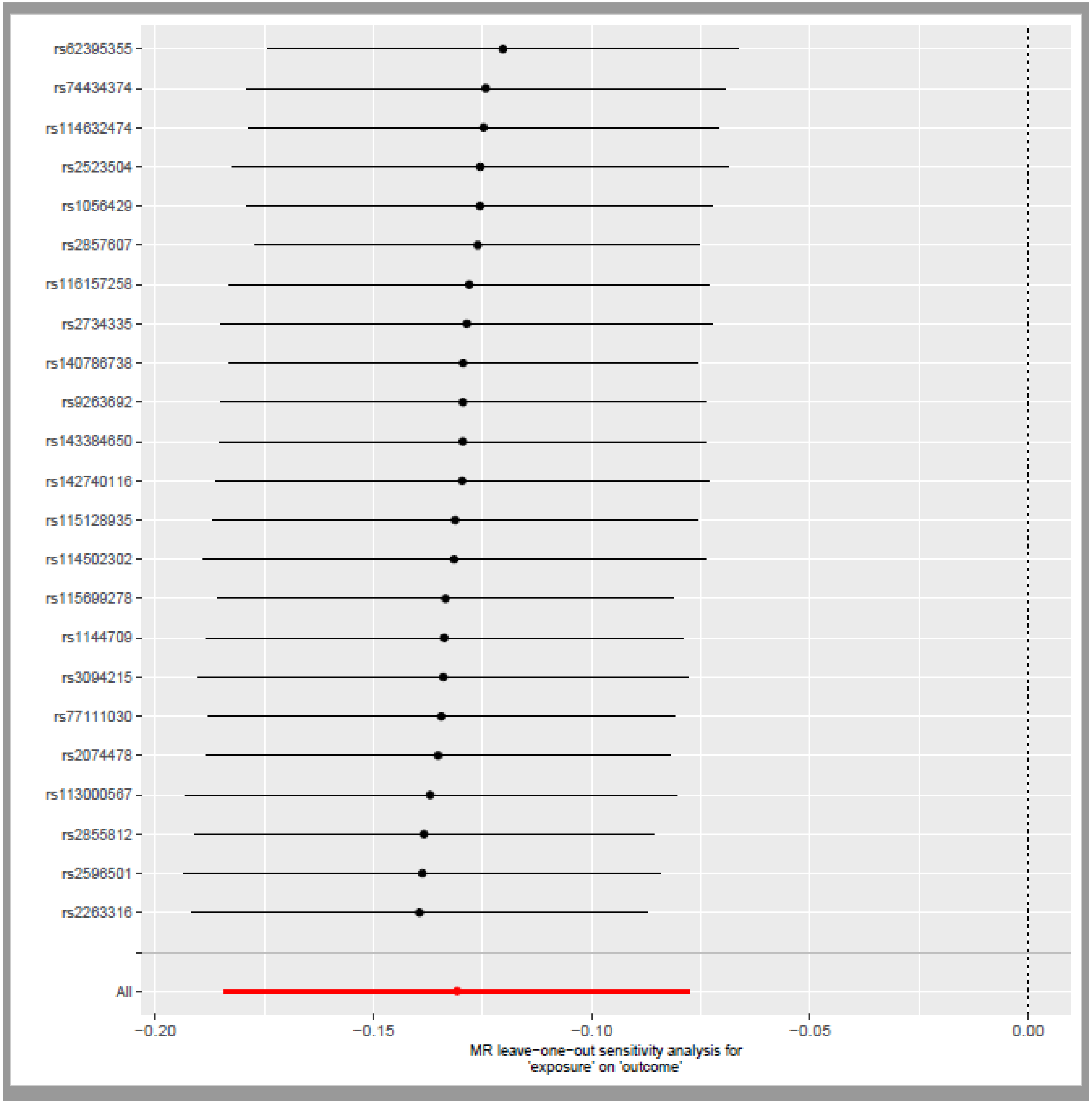
rs62395355
rs74434374
rs114632474
rs2523504
rs1056429
rs2857607
rs116157258
rs2734335
rs140786738
rs9263692
rs143384650
rs142740116
rs115128935
rs114502302
rs115699278
rs1144709
rs3094215
rs77111030
rs2074478
rs113000567
rs2855812
rs2596501
rs2263316
All
−0.20
−0.15
−0.10
−0.05
0.00
MR leave−one−out sensitivity analysis for
'exposure' on 'outcome'

NMB

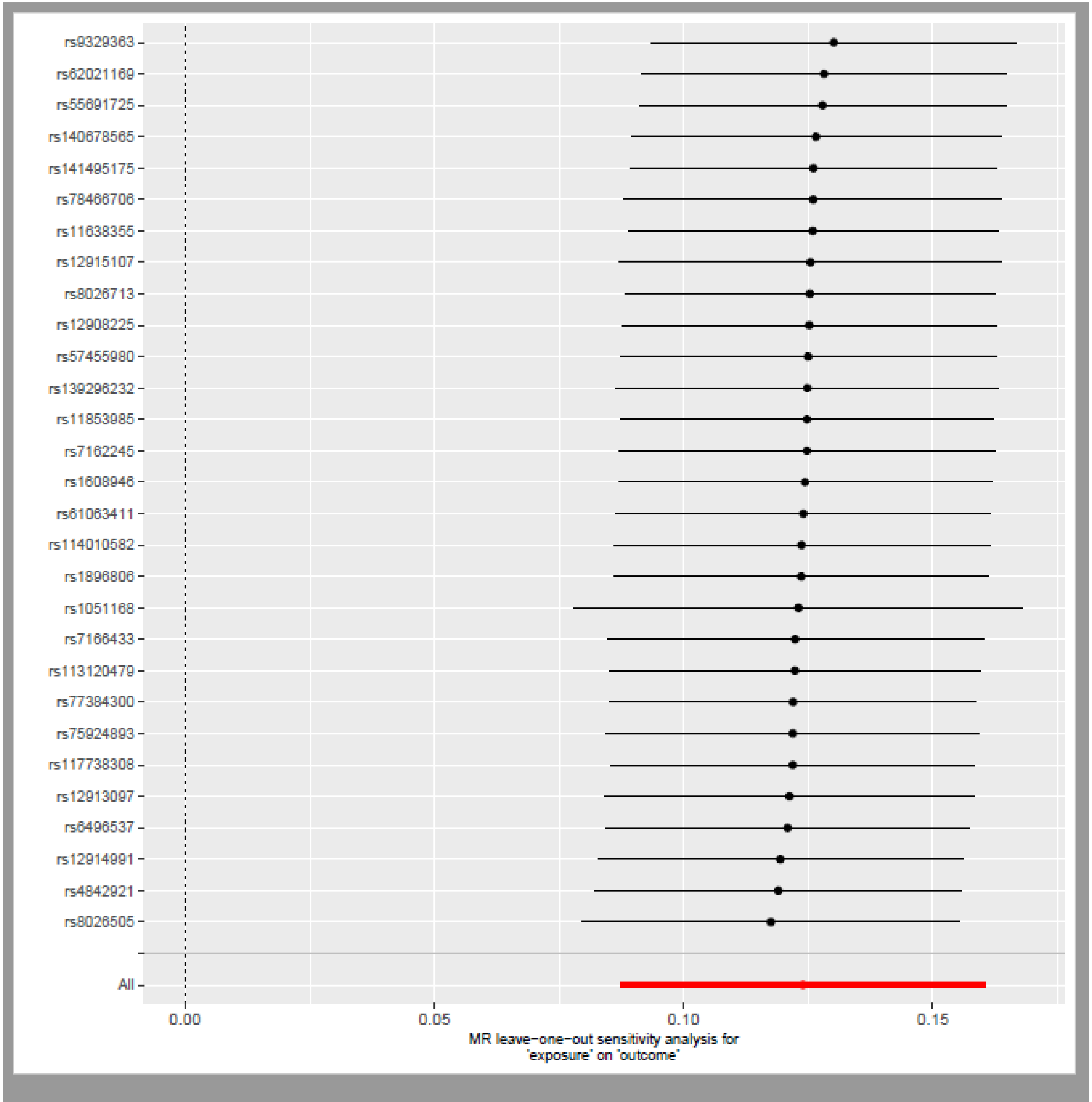
rs9329363
rs62021169
rs55691725
rs140678565
rs141495175
rs78466706
rs11638355
rs12915107
rs8026713
rs12908225
rs57455980
rs139296232
rs11853985
rs7162245
rs1608946
rs61063411
rs114010582
rs1896806
rs1051168
rs7166433
rs113120479
rs77384300
rs75924893
rs117738308
rs12913097
rs6496537
rs12914991
rs4842921
rs8026505
All
0.00
0.05
0.10
0.15
MR leave−one−out sensitivity analysis for
'exposure' on 'outcome'

NUCB2

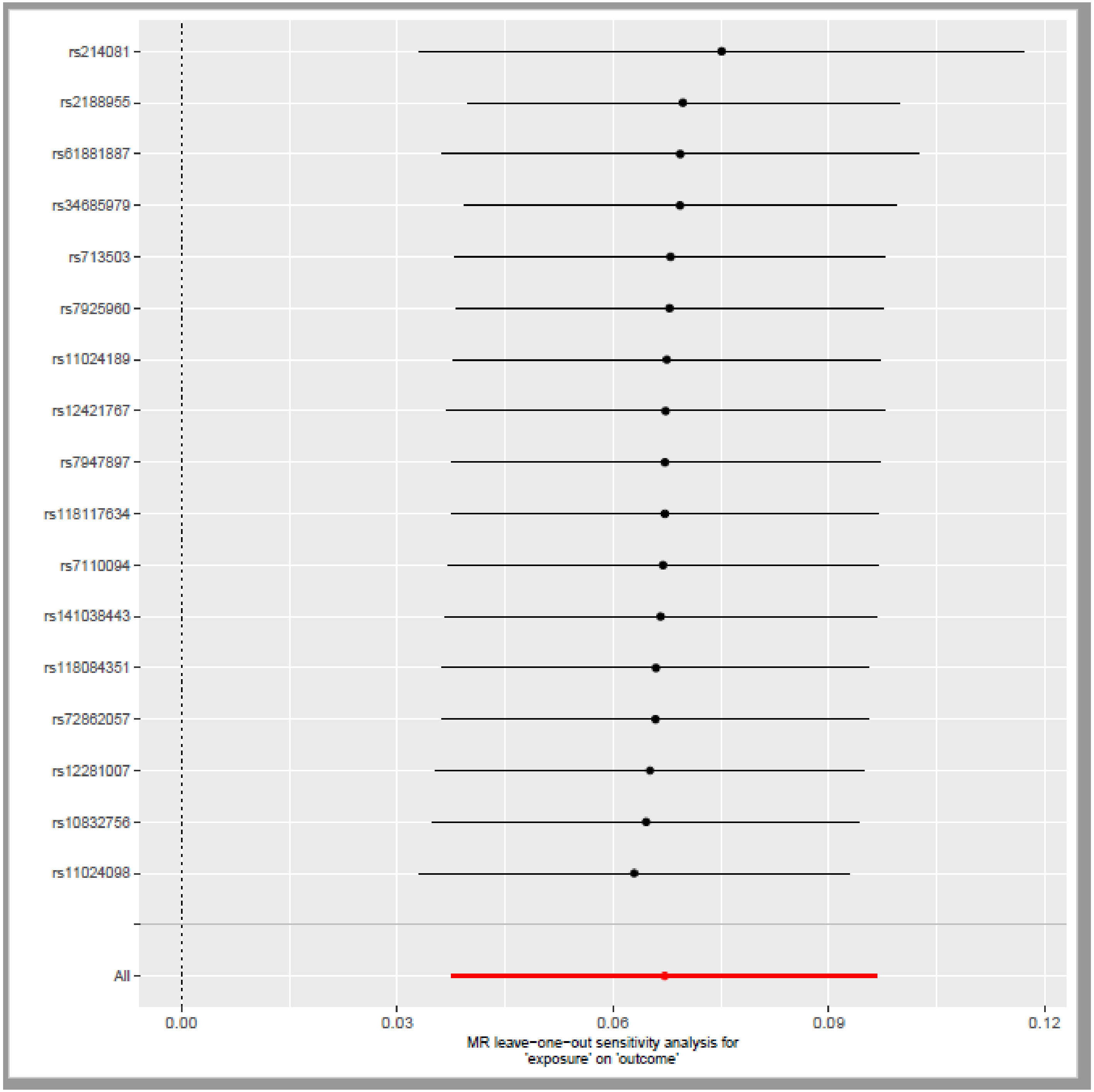
rs214081
rs2188955
rs61881887
rs34685979
rs713503
rs7925960
rs11024189
rs12421767
rs7947897
rs118117634
rs7110094
rs141038443
rs118084351
rs72862057
rs12281007
rs10832756
rs11024098
All
0.00
0.03
0.06
0.09
0.12
MR leave-one-out sensitivity analysis for
'exposure' on 'outcome'

PLK1

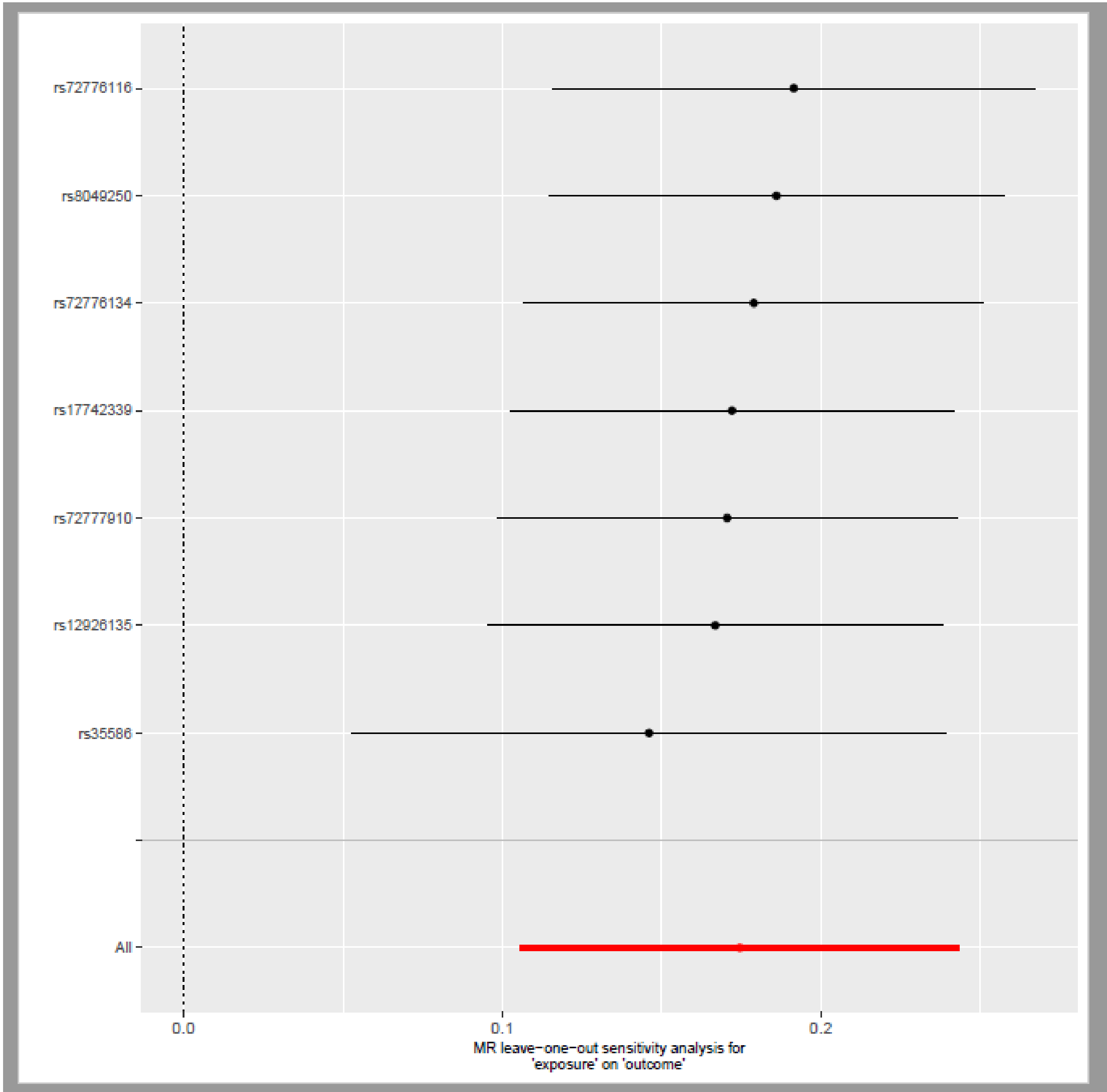
rs72776116
rs8049250
rs72776134
rs17742339
rs72777810
rs12926135
rs35586
All
0.0
0.1
0.2
MR leave−one−out sensitivity analysis for
'exposure' on 'outcome'

PPIF

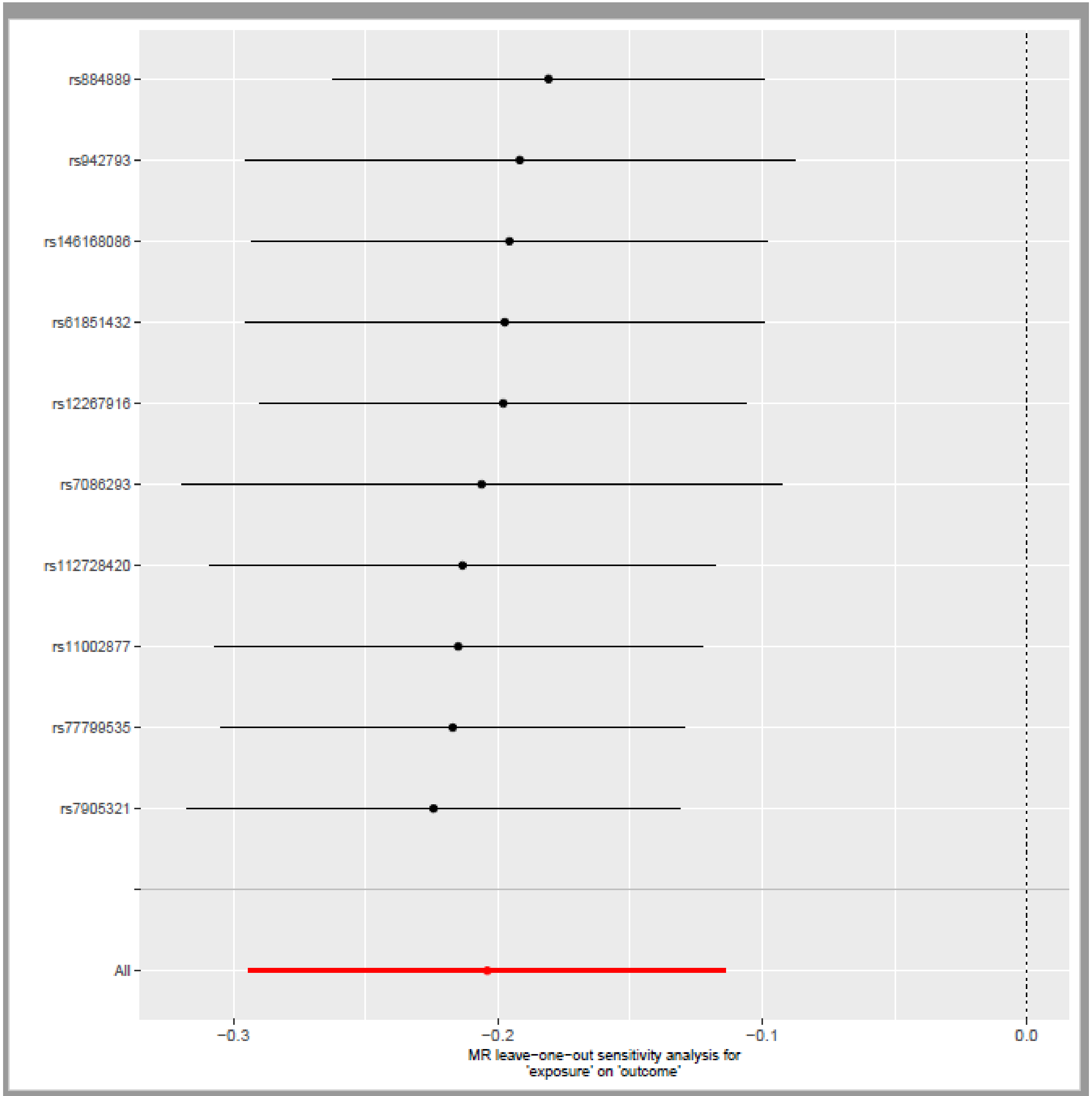

rs884889
rs942793
rs146168086
rs61851432
rs12267916
rs7086293
rs112728420
rs11002877
rs77799535
rs7905321
All
−0.3
−0.2
−0.1
0.0
MR leave−one−out sensitivity analysis for
'exposure' on 'outcome'

PPIL3

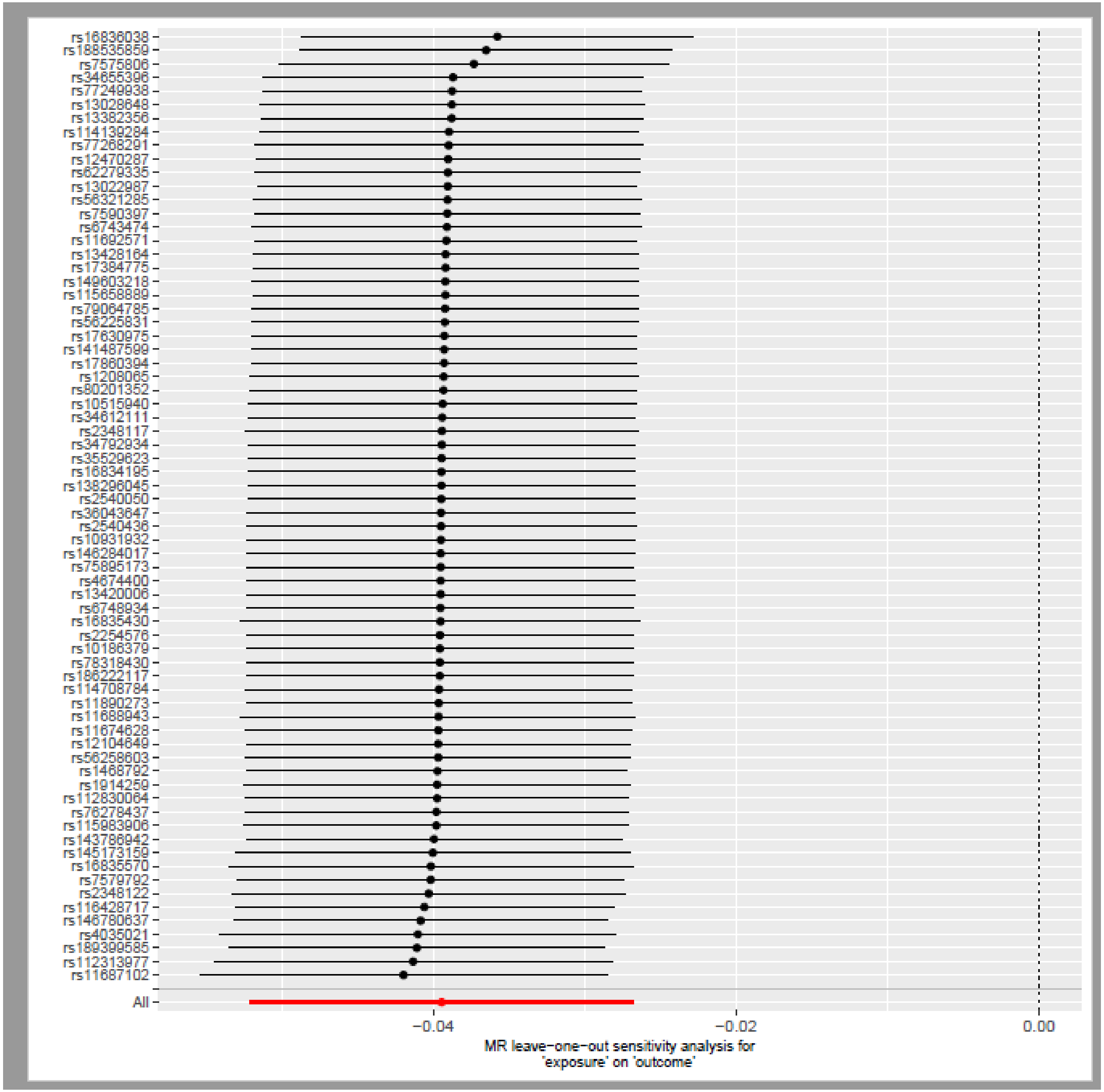

rs16836038
rs188535859
rs7575806
rs34655396
rs77249938
rs13028648
rs13382356
rs114139284
rs77268291
rs12470287
rs62279335
rs13022987
rs56321285
rs7590397
rs6743474
rs11692571
rs13428164
rs17384775
rs149603218
rs115658889
rs79064785
rs56225831
rs17630975
rs141487599
rs17860394
rs1208065
rs80201352
rs10515940
rs34612111
rs2348117
rs34792934
rs35529623
rs16834195
rs138296045
rs2540050
rs36043647
rs2540436
rs10931932
rs146284017
rs75895173
rs4674400
rs13420006
rs6748934
rs16835430
rs2254576
rs10186379
rs78318430
rs186222117
rs114708784
rs11890273
rs11688943
rs11674628
rs12104649
rs56258603
rs1468792
rs1914259
rs112830064
rs76278437
rs115983906
rs143786942
rs145173159
rs16835570
rs7579792
rs2348122
rs116428717
rs146780637
rs4035021
rs189399585
rs112313977
rs11687102
All
−0.04
−0.02
0.00
MR leave−one−out sensitivity analysis for
'exposure' on 'outcome'

PPP5C

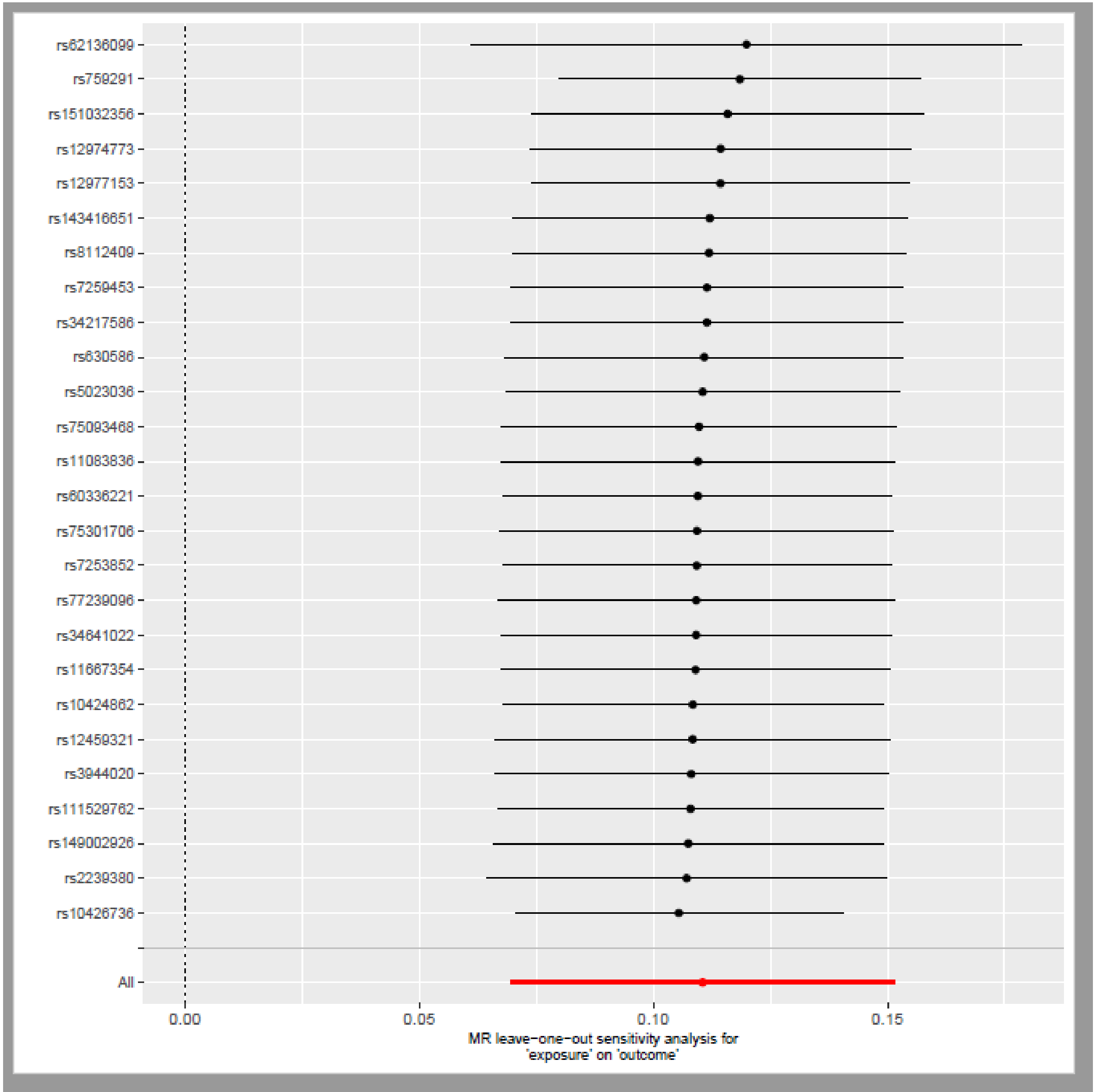

rs62136099
rs759291
rs151032356
rs12974773
rs12977153
rs143416651
rs8112409
rs7259453
rs34217586
rs630586
rs5023036
rs75093468
rs11083836
rs60336221
rs75301706
rs7253852
rs77239096
rs34641022
rs11667354
rs10424862
rs12459321
rs3944020
rs111529762
rs149002926
rs2239380
rs10426736
All
0.00
0.05
0.10
0.15
MR leave−one−out sensitivity analysis for
'exposure' on 'outcome'

PRKCQ

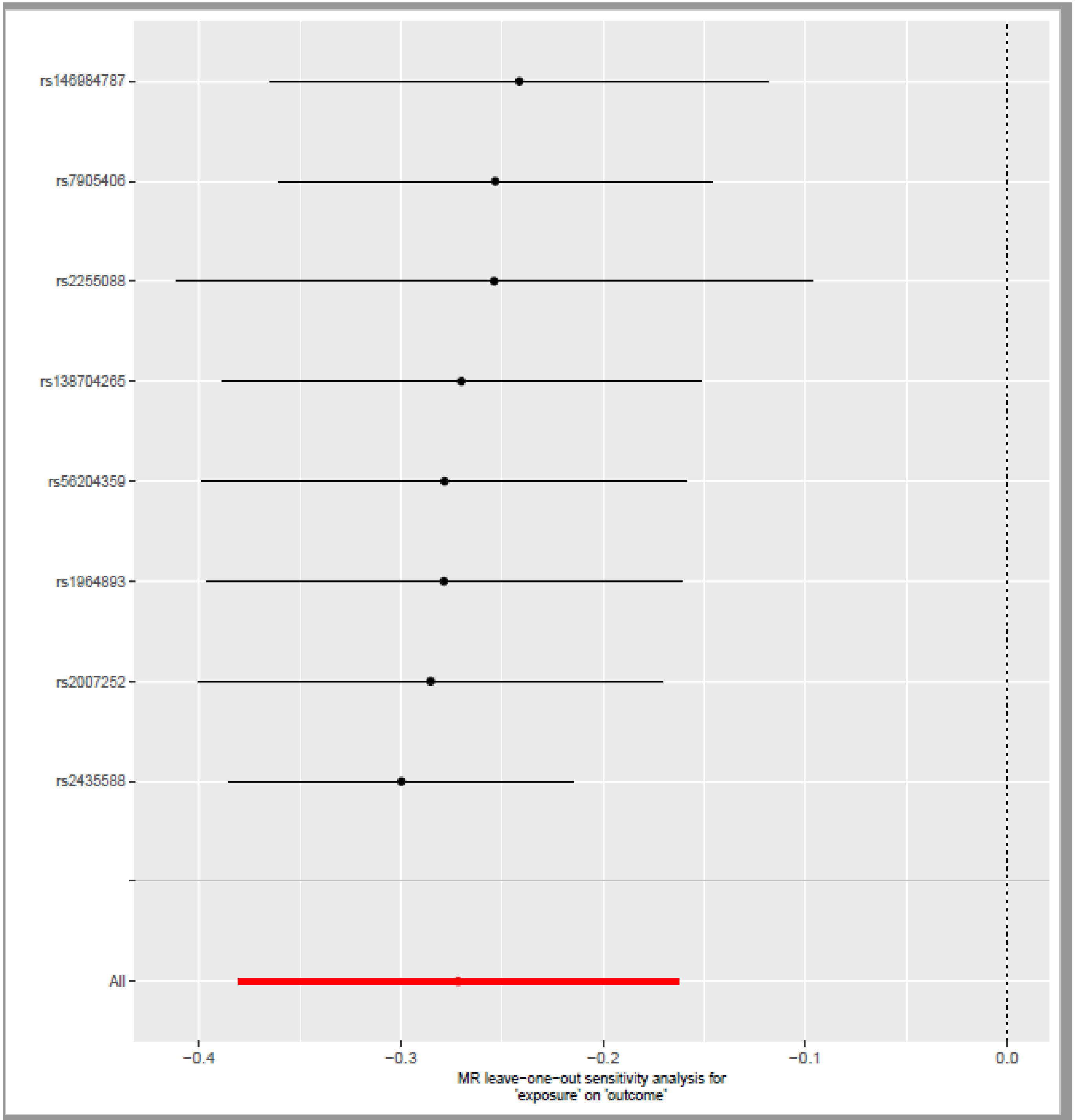
rs146984787
rs7905406
rs2255088
rs138704265
rs56204359
rs1964893
rs2007252
rs2435588
All
−0.4
−0.3
−0.2
−0.1
0.0
MR leave−one−out sensitivity analysis for
'exposure' on 'outcome'

PSEN2

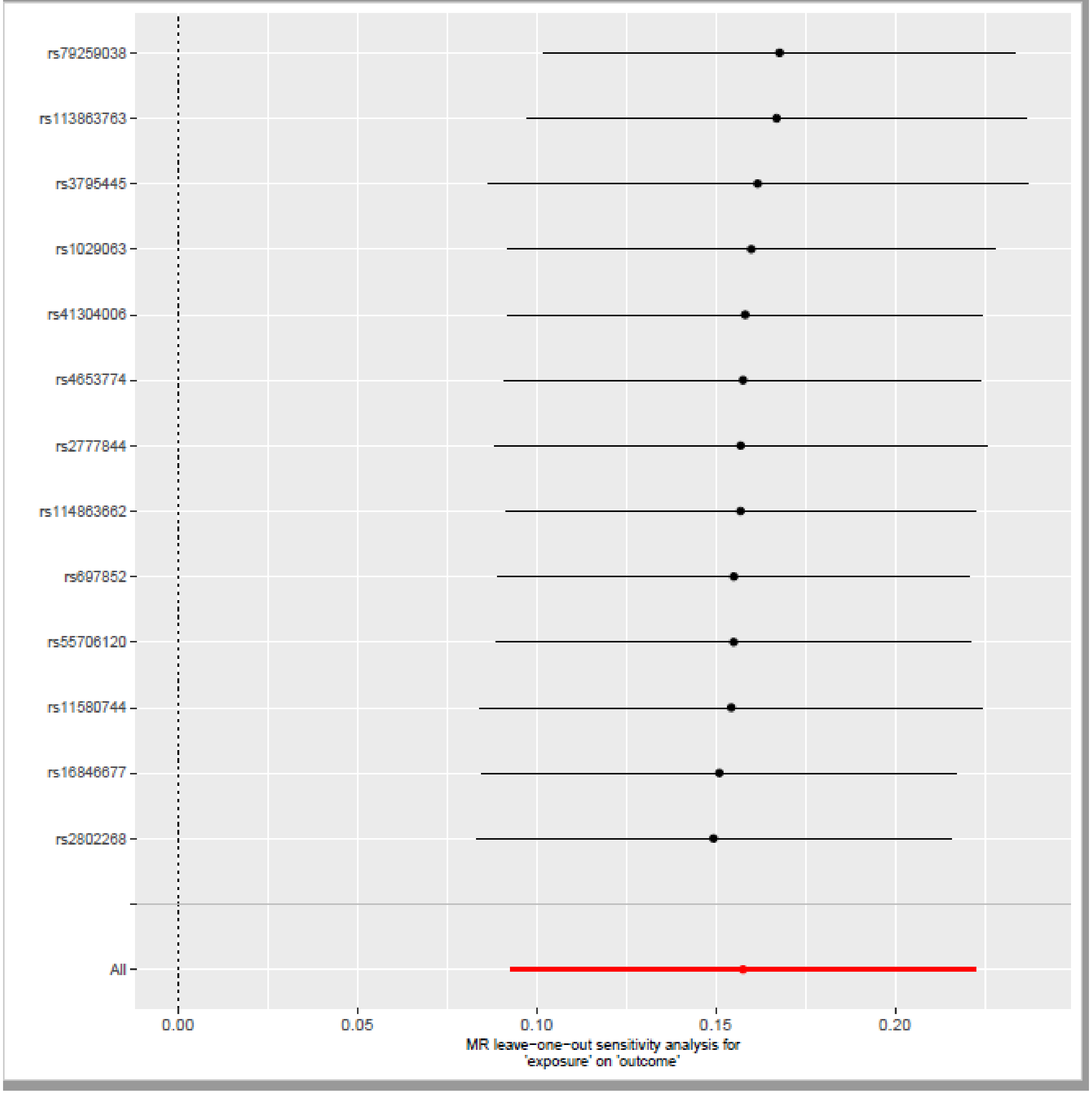

rs79259038
rs113863763
rs3795445
rs1029063
rs41304006
rs4653774
rs2777844
rs114863662
rs697852
rs55706120
rs11580744
rs16846677
rs2802268
All
0.00
0.05
0.10
0.15
0.20
MR leave−one−out sensitivity analysis for
'exposure' on 'outcome'

SENP7

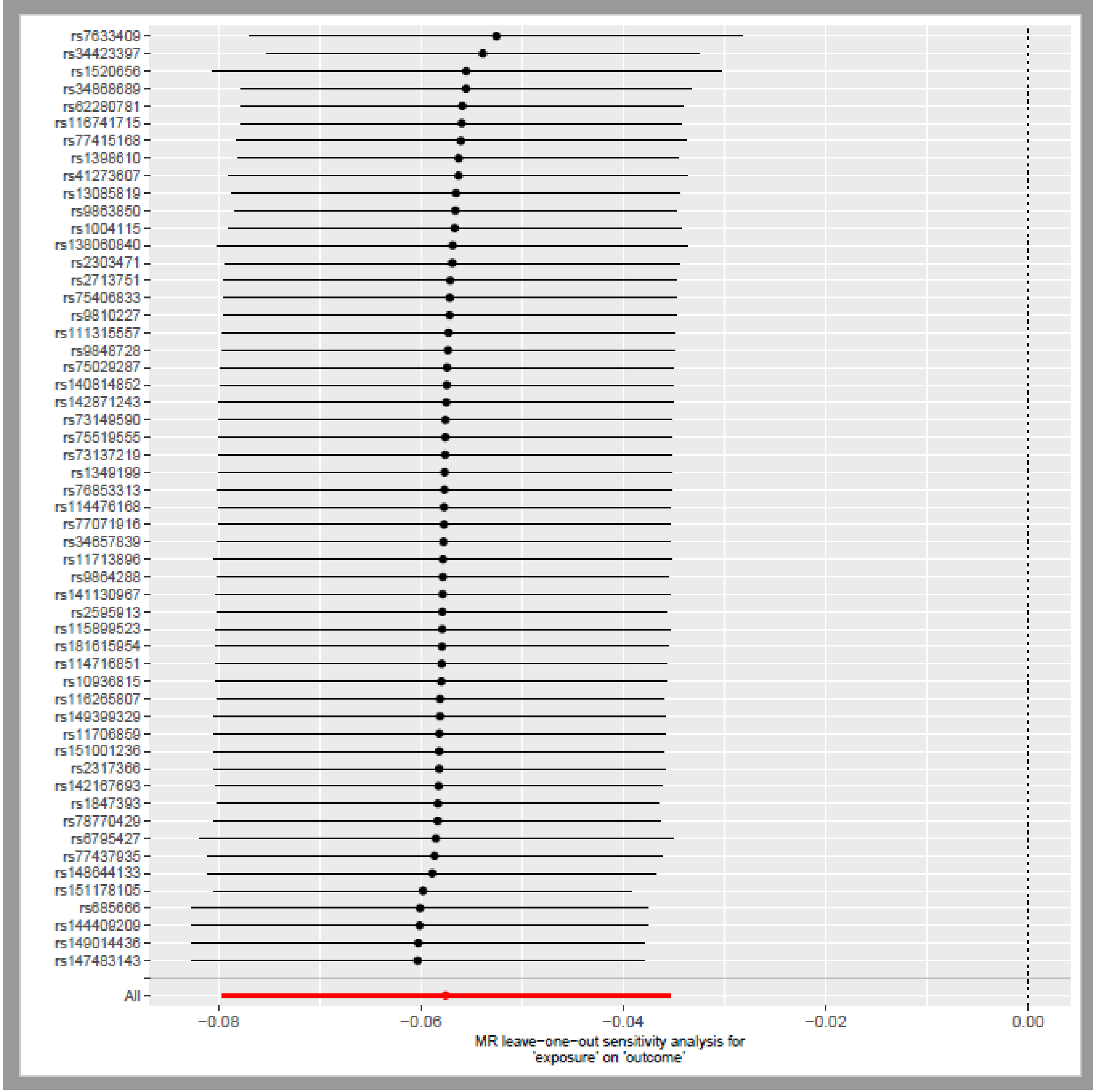

rs7633409
rs34423397
rs1520656
rs34868689
rs62280781
rs116741715
rs77415168
rs1398610
rs41273607
rs13085819
rs9863850
rs1004115
rs138060840
rs2303471
rs2713751
rs75406833
rs9810227
rs111315557
rs9848728
rs75029287
rs140814852
rs142871243
rs73149590
rs75519555
rs73137219
rs1349199
rs76853313
rs114476168
rs77071916
rs34657839
rs11713896
rs9864288
rs141130967
rs2595913
rs115899523
rs181615954
rs114716851
rs10936815
rs116265807
rs149399329
rs11706859
rs151001236
rs2317366
rs142167693
rs1847393
rs78770429
rs6795427
rs77437935
rs148644133
rs151178105
rs685666
rs144409209
rs149014436
rs147483143
All
−0.08
−0.06
−0.04
−0.02
0.00
MR leave−one−out sensitivity analysis for
'exposure' on 'outcome'

SLC22A4

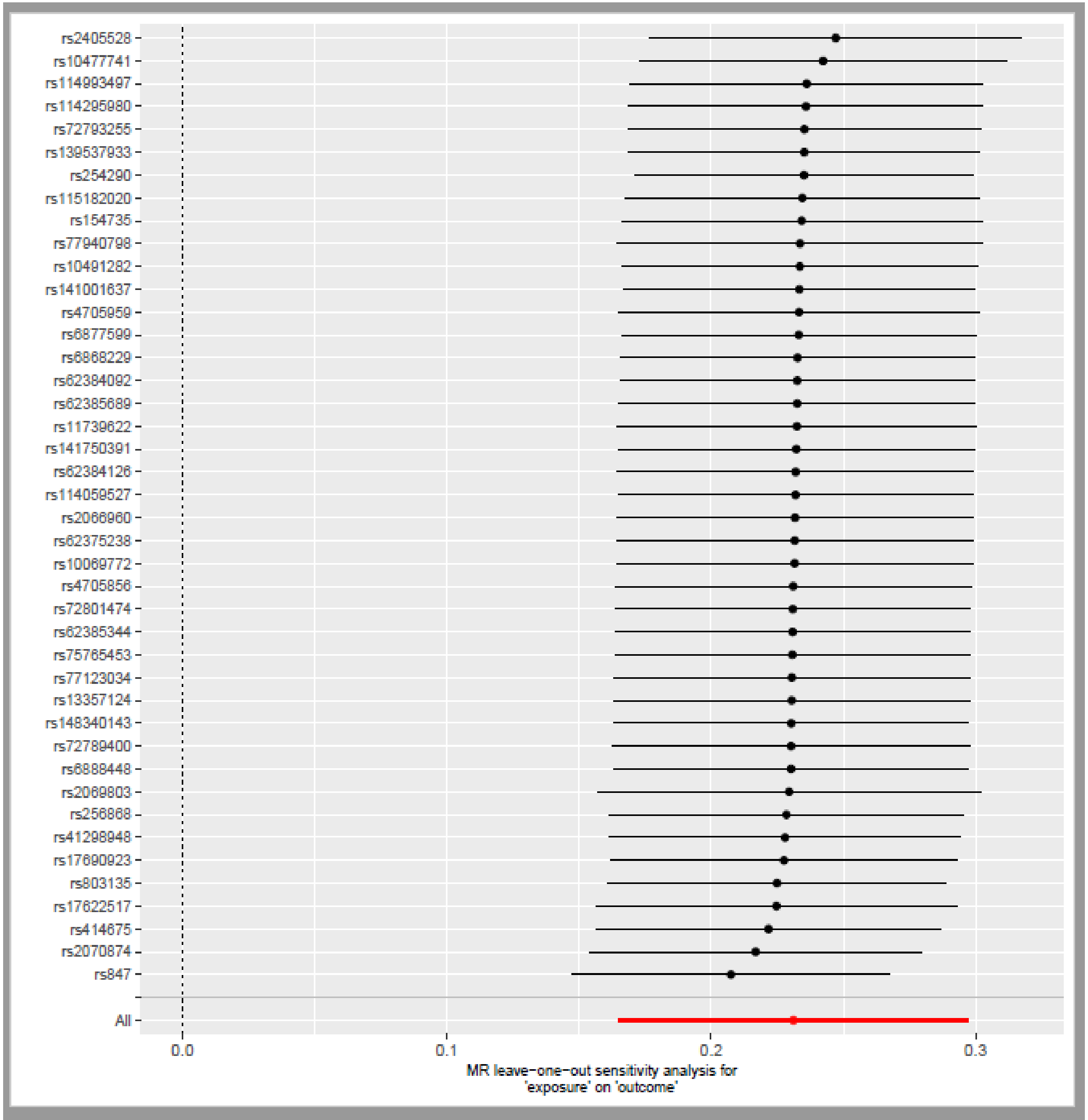
rs2405528
rs10477741
rs114993497
rs114295980
rs72793255
rs139537933
rs254290
rs115182020
rs154735
rs77940798
rs10491282
rs141001637
rs4705959
rs6877599
rs6868229
rs62384092
rs62385689
rs11739622
rs141750391
rs62384126
rs114059527
rs2066960
rs62375238
rs10069772
rs4705856
rs72801474
rs62385344
rs75765453
rs77123034
rs13357124
rs148340143
rs72789400
rs6888448
rs2069803
rs256868
rs41298948
rs17690923
rs803135
rs17622517
rs414675
rs2070874
rs847
All
0.0
0.1
0.2
0.3
MR leave−one−out sensitivity analysis for
'exposure' on 'outcome'

SLC22A5

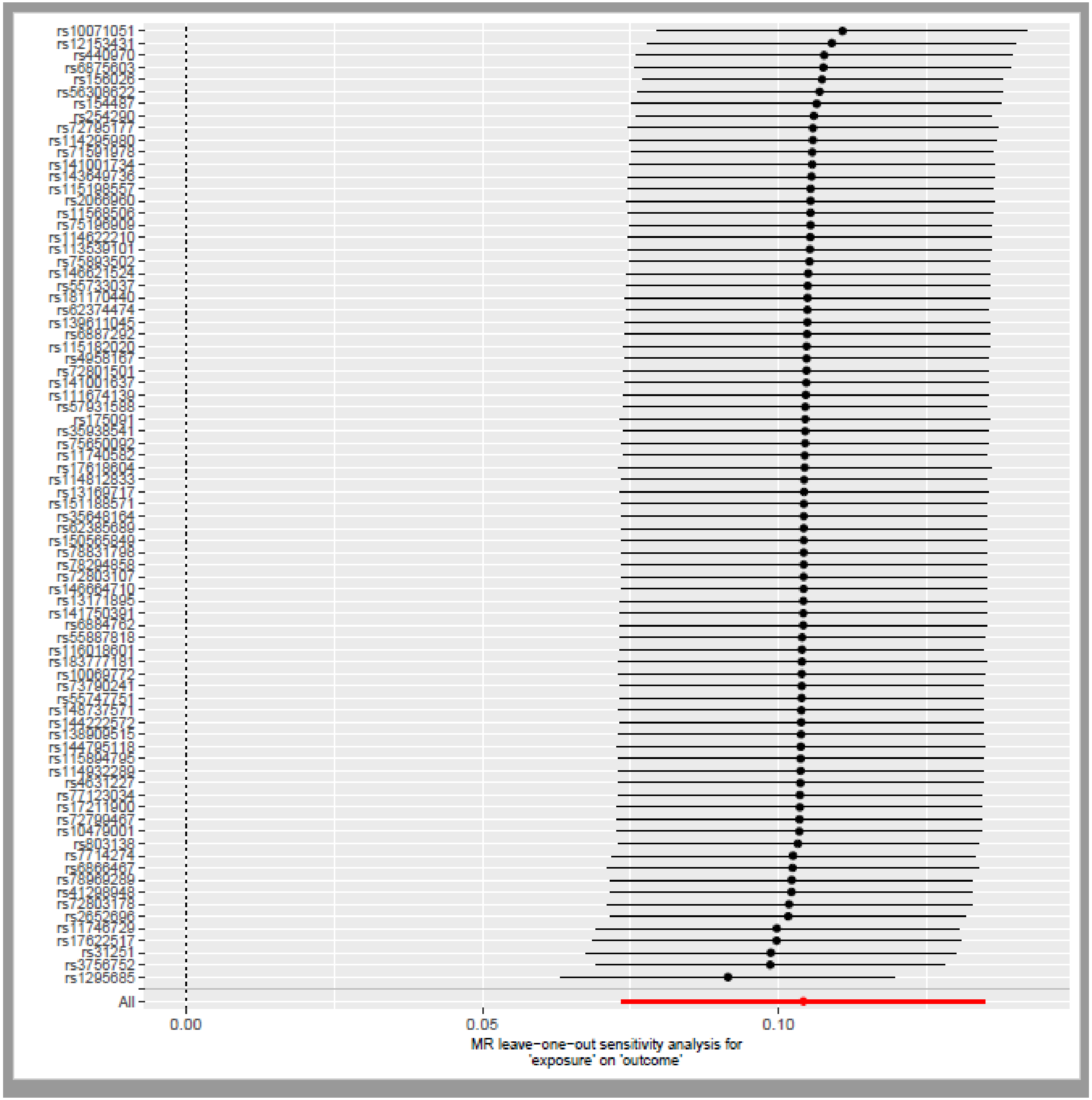

All
0.00
0.05
0.10
MR leave−one−out sensitivity analysis for
'exposure' on 'outcome'

SLK

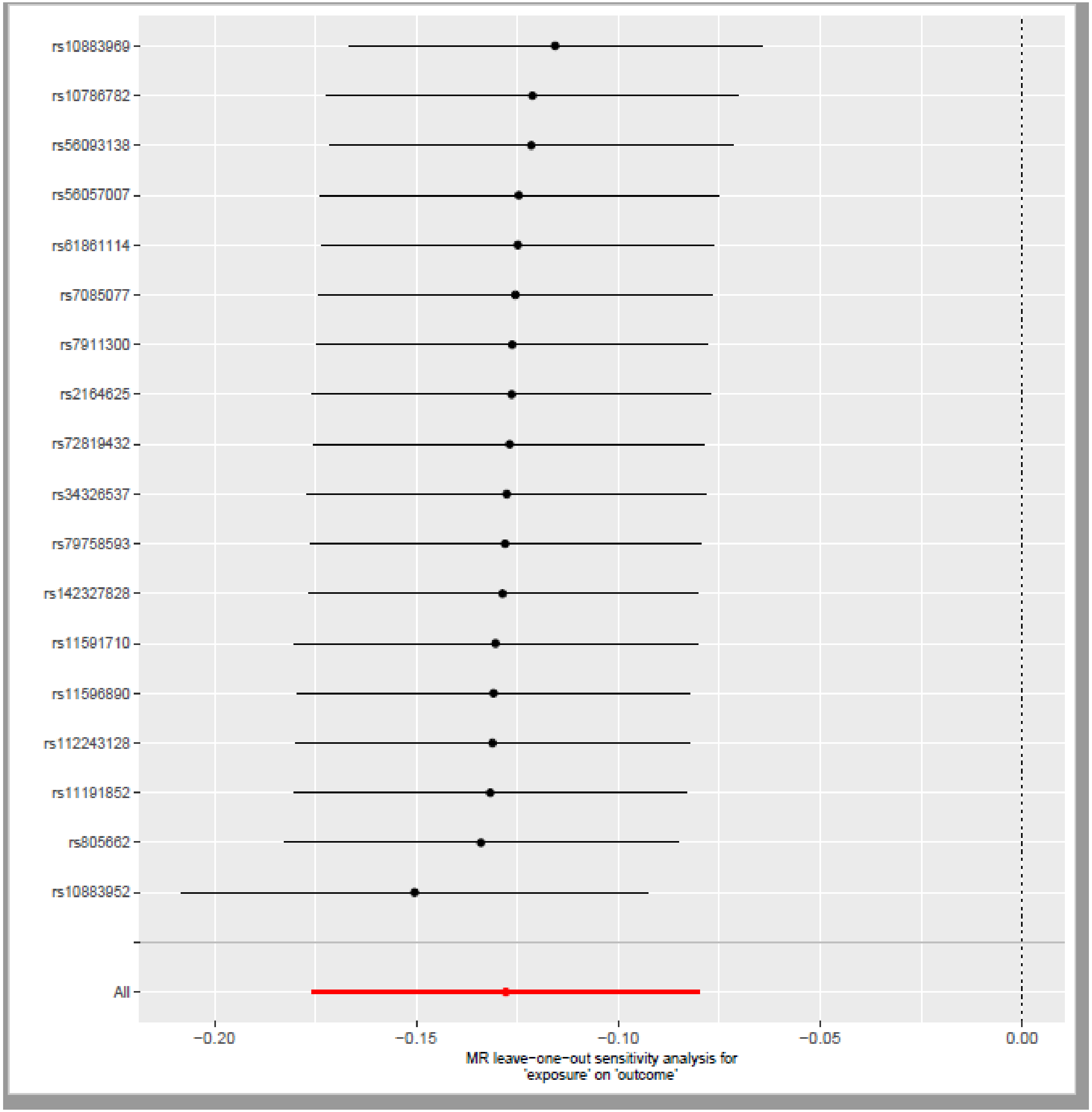
rs10883969
rs10786782
rs56093138
rs56057007
rs61861114
rs7085077
rs7911300
rs2164625
rs72819432
rs34326537
rs79758593
rs142327828
rs11591710
rs11596890
rs112243128
rs11191852
rs805662
rs10883952
All
−0.20
−0.15
−0.10
−0.05
0.00
MR leave−one−out sensitivity analysis for
'exposure' on 'outcome'

TAPBPL

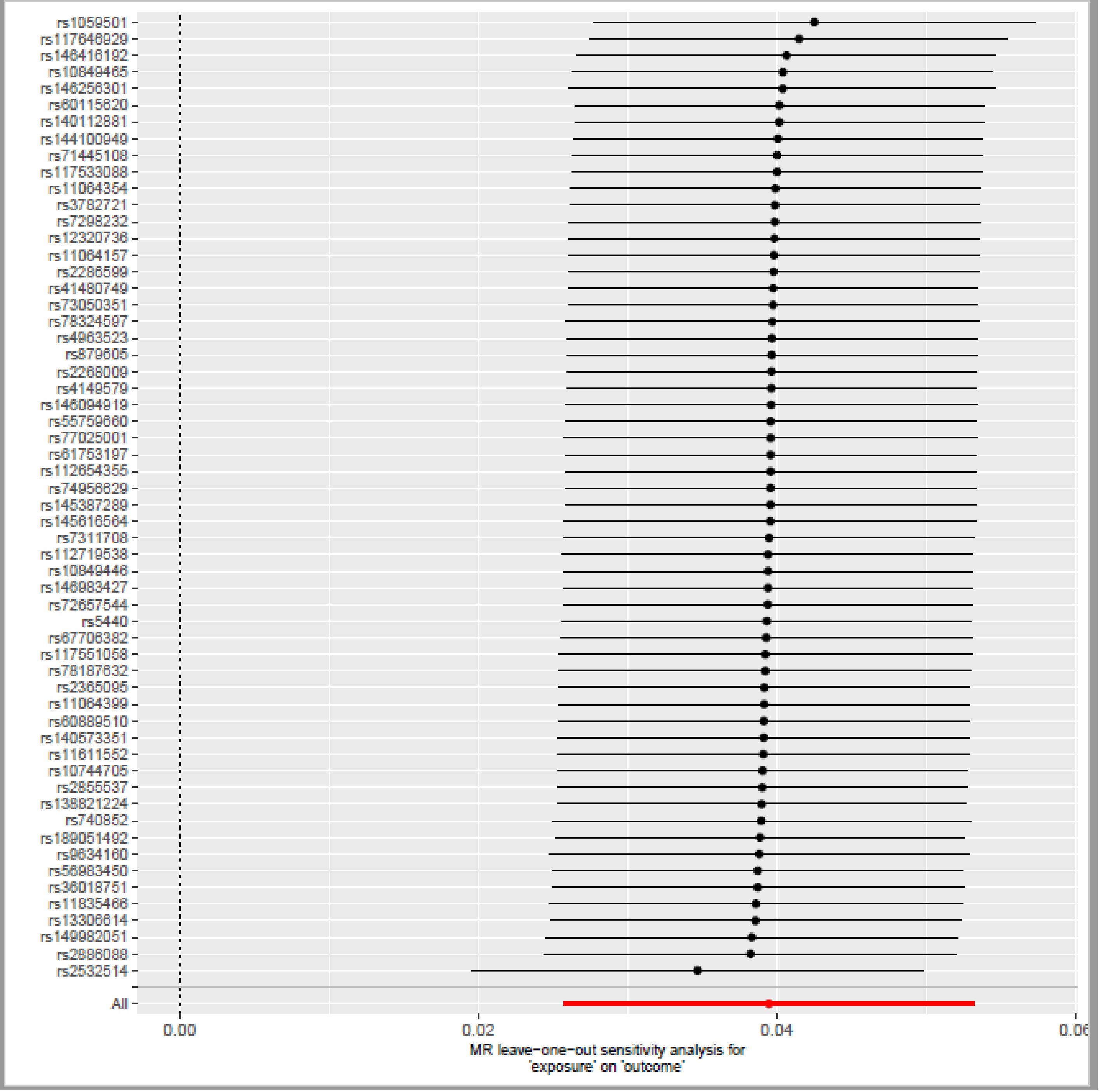

rs1059501
rs117646929
rs146416192
rs10849465
rs146256301
rs60115620
rs140112881
rs144100949
rs71445108
rs117533088
rs11064354
rs3782721
rs7298232
rs12320736
rs11064157
rs2286599
rs41480749
rs73050351
rs78324597
rs4963523
rs879605
rs2268009
rs4149579
rs146094919
rs55759660
rs77025001
rs61753197
rs112654355
rs74956629
rs145387289
rs145616564
rs7311708
rs112719538
rs10849446
rs146983427
rs72657544
rs5440
rs67706382
rs117551058
rs78187632
rs2365095
rs11064399
rs60889510
rs140573351
rs11611552
rs10744705
rs2855537
rs138821224
rs740852
rs189051492
rs9634160
rs56983450
rs36018751
rs11835466
rs13306614
rs149982051
rs2886088
rs2532514
All
0.00
0.02
0.04
0.06
MR leave−one−out sensitivity analysis for
'exposure' on 'outcome'

TG

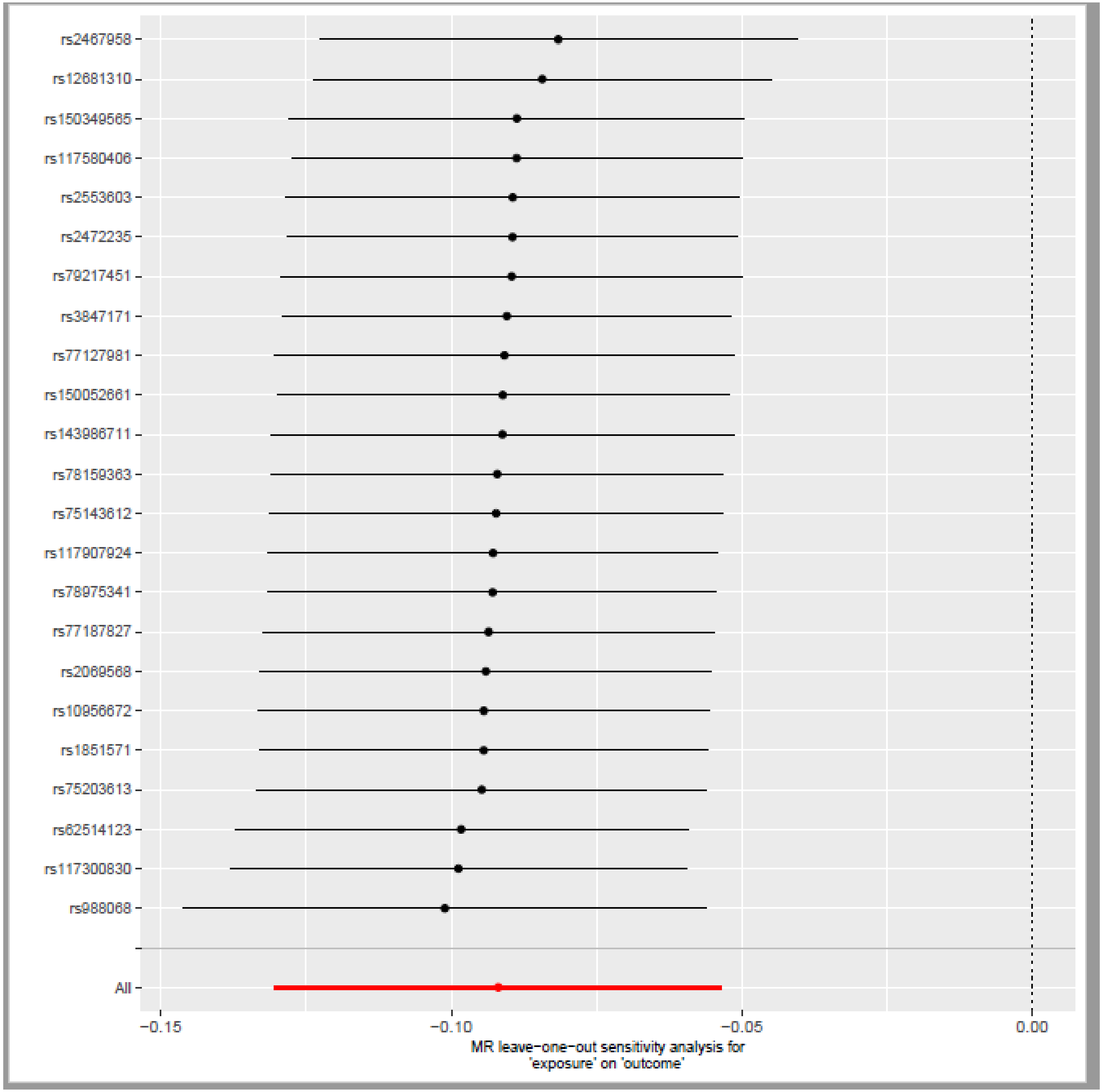

rs2467958
rs12681310
rs150349565
rs117580406
rs2553603
rs2472235
rs79217451
rs3847171
rs77127981
rs150052661
rs143986711
rs78159363
rs75143612
rs117907924
rs78975341
rs77187827
rs2069568
rs10956672
rs1851571
rs75203613
rs62514123
rs117300830
rs988068
All
−0.15
−0.10
−0.05
0.00
MR leave−one−out sensitivity analysis for
'exposure' on 'outcome'

TNFRSF 14

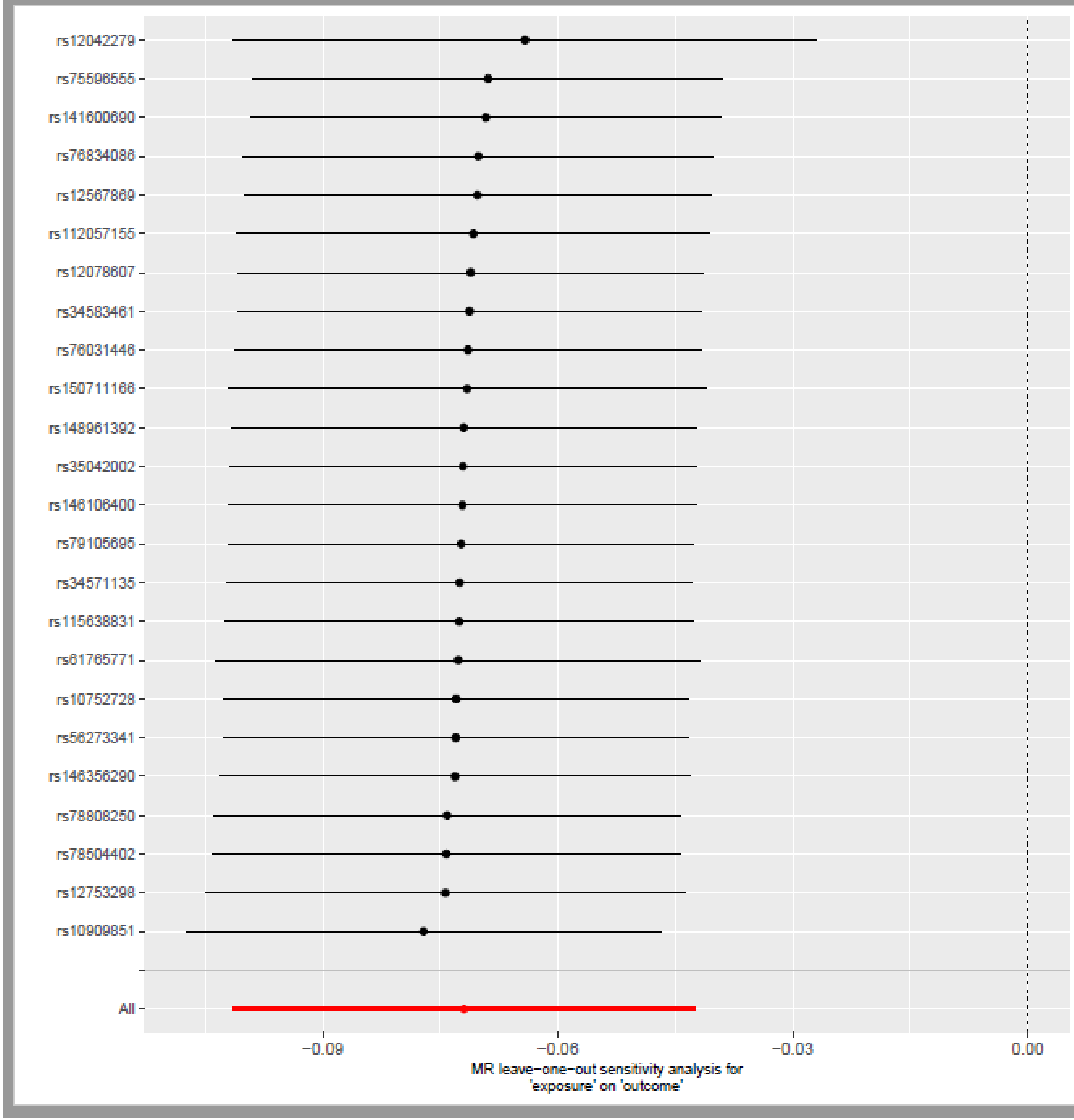
rs12042279
rs75596555
rs141600690
rs76834086
rs12567869
rs112057155
rs12078607
rs34583461
rs76031446
rs150711166
rs148961392
rs35042002
rs146106400
rs79105695
rs34571135
rs115638831
rs61765771
rs10752728
rs56273341
rs146356290
rs78808250
rs78504402
rs12753298
rs10909851
All
−0.09
−0.06
−0.03
0.00
MR leave−one−out sensitivity analysis for
'exposure' on 'outcome'

VAMP1

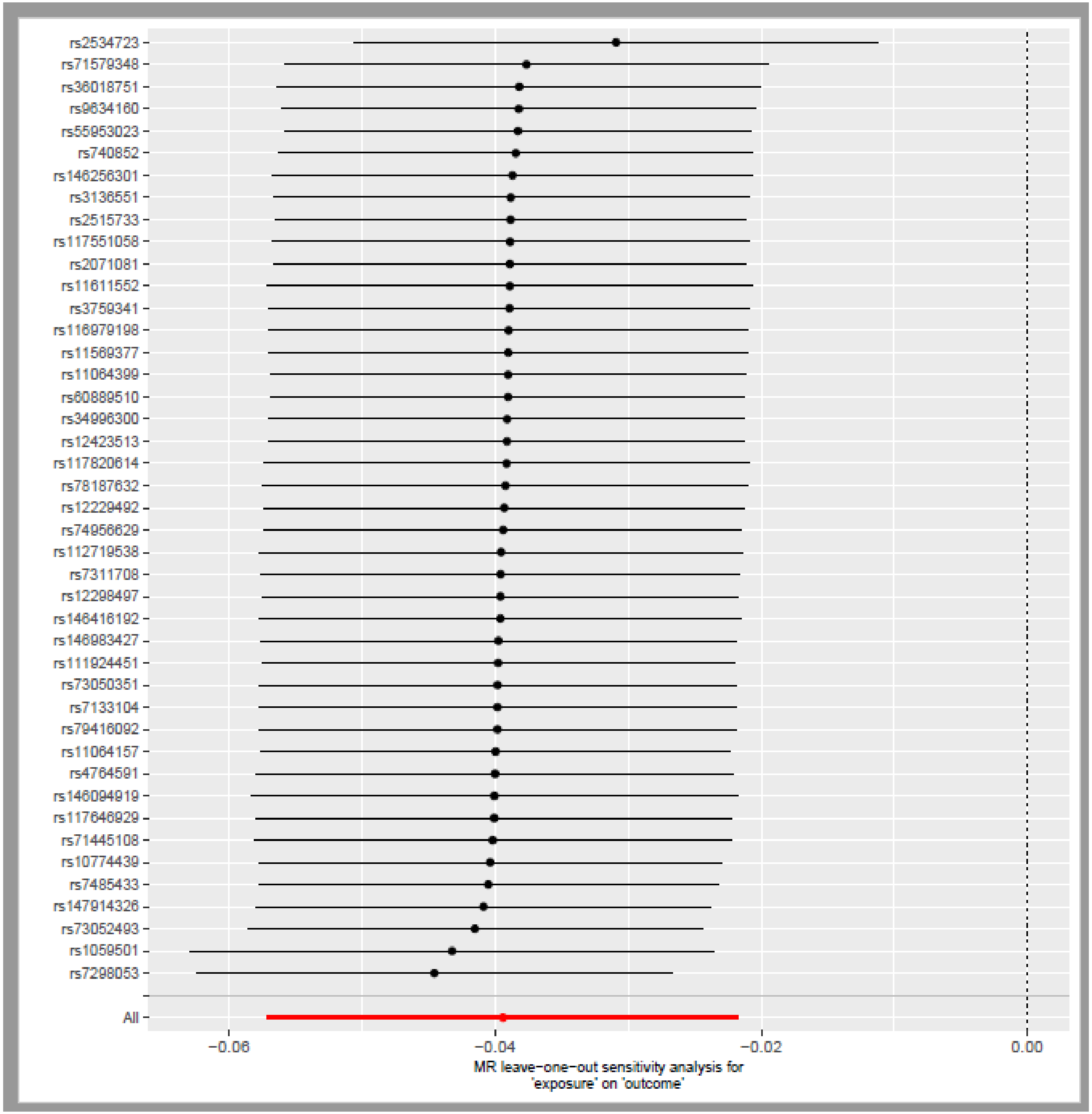
rs2534723
rs71579348
rs36018751
rs9634160
rs55953023
rs740852
rs146256301
rs3136551
rs2515733
rs117551058
rs2071081
rs11611552
rs3759341
rs116979198
rs11569377
rs11064399
rs60889510
rs34996300
rs12423513
rs117820614
rs78187632
rs12229492
rs74956629
rs112719538
rs7311708
rs12298497
rs146416192
rs146983427
rs111924451
rs73050351
rs7133104
rs79416092
rs11064157
rs4764591
rs146094919
rs117646929
rs71445108
rs10774439
rs7485433
rs147914326
rs73052493
rs1059501
rs7298053
All
−0.06
−0.04
−0.02
0.00
MR leave−one−out sensitivity analysis for
'exposure' on 'outcome'
